# Key features and guidelines for the application of microbial alpha diversity metrics

**DOI:** 10.1038/s41598-024-77864-y

**Published:** 2025-01-03

**Authors:** Ignacio Cassol, Mauro Ibañez, Juan Pablo Bustamante

**Affiliations:** 1 Facultad de Ingeniería, Universidad Austral, LIDTUA, CIC, Buenos Aires, Argentina; 2 Facultad de Ingeniería, Universidad Nacional de Entre Ríos, Oro Verde, Argentina; 3 CONICET, Buenos Aires, Argentina

**Keywords:** Microbiome, Data processing, Standards, Biodiversity

## Abstract

Studies of microbial communities vary widely in terms of analysis methods. In this growing field, the wide variety of diversity measures and lack of consistency make it harder to compare different studies. Most existing alpha diversity metrics are inherited from other disciplines and their assumptions are not always directly meaningful or true for microbiome data. Many existing microbiome studies apply one or some alpha diversity metrics with no fundamentals but also an unclear results interpretation. This work focuses on a theoretical, empirical, and comparative analysis of 19 frequently and less-frequently used microbial alpha diversity metrics grouped into 4 proposed categories, including key features of every analyzed metric with their mathematical assumptions, to provide a deeper understanding of the existing metrics and a practical implementation guide for future studies. Key metrics that should be required in microbiome analysis include richness, phylogenetic diversity, entropy, dominance of a few microbes over others, and an estimate of unobserved microbes. Collectively, these metrics contribute to a comprehensive set of analyses characterizing samples, allowing the determination of key aspects that might be otherwise obscured by partial or biased information. These guidelines enable further detailed analysis by each author according to their specific interests and clinical trials. Several practical examples are provided to illustrate how these recommendations improve the quality and depth of information obtained, facilitating better interpretation when working with microbiome data. These guidelines can be applied to both existing and future research studies, enhancing the standardization, consistency, and robustness of the analyses conducted. This approach aims to improve the capture of biological diversity, leading to better interpretations and insights.

**Supplementary Information:**

The online version contains supplementary material available at 10.1038/s41598-024-77864-y.

## Introduction

The analysis of the composition of microbial communities has led to better insights in the roles that microbes play in human health^1–3^, animal health, ecosystems, and agrotech niches^4,5^. However, technical, and procedural issues are preventing this from being possible in a growing field without a clear characterization of technical features of currently used metrics. Because microbes have tremendous potential to impact our physiology, our environment, both in health and disease, and even whole ecosystems, a precise and reproducible characterization of each microbiome’s composition is fundamental^6,7^. Given that the microbiota composition is related to the host health^8–10^, the study of its intrinsic diversity, including the microorganisms’ richness and their distribution present in a sample has been usually included in every published study.

Two common measures reported in many microbiome studies are alpha- and beta-diversity. Alpha diversity is a general term for metrics that describe the species richness, evenness, or diversity within a sample. In contrast, beta-diversity measures compare the similarity of two or more communities^11^. Although the term alpha diversity is widely used, it is an ambiguous concept since it encompasses several complementary aspects, including the number of microorganisms, the distribution of their abundances, and their phylogenetic relationship. These aspects could be taken into consideration separately or combined, but are not as synonymous as they are usually used.

Most existing alpha diversity metrics are inherited from other disciplines, such as plant or insect biodiversity studies^12,13^ and their assumptions are not always directly meaningful or true for microbiome data. In addition, the measurement units of some metrics have elusive or not easily understandable biological meanings, being difficult to interpret, and neither translatable nor comparable. Given this basis, many existing microbiome studies apply one or some alpha diversity metrics with no fundamentals but also without a clear results interpretation.

Due to the relative novelty of microbiome studies as a discipline, there is a lack of consensus and standardization processes among researchers and clinicians. This creates significant challenges in obtaining, analyzing, visualizing, interpreting, and comparing human microbiome data robustly, particularly as these practices move towards clinical applications^14–16^. This work focuses on a theoretical and comparative analysis of several alpha diversity metrics used in microbiome analysis, detailing the key features and mathematical assumptions of each metric, to provide a deeper understanding of these metrics and offer a practical implementation guide for future studies.

## Results

Alpha metrics used in 68 microbiome studies or in the QIIME 2 suite were grouped into four categories after performing a detailed theoretical analysis of each metric’s mathematical formula (see Methods section for a detailed explanation). The proposed categories with their associated metrics are:

- Richness: Chao1, ACE, Fisher, Margalef, Menhinick, Observed, and Robbins.
- Dominance (also known as evenness): Berger-Parker, Dominance, Simpson, ENSPIE, Gini, McIntosh, and Strong.
- Phylogenetics: Faith.
- Information: Shannon, Brillouin, Heip and Pielou.

The 19 selected alpha diversity metrics were calculated for the sequence datasets obtained for a total of 4,596 stool samples included in 13 publicly available human microbiome projects (Table S3). All sequence data were reanalyzed for this paper using the same data processing pipeline, as described in Methods.

**Fig. 1:**
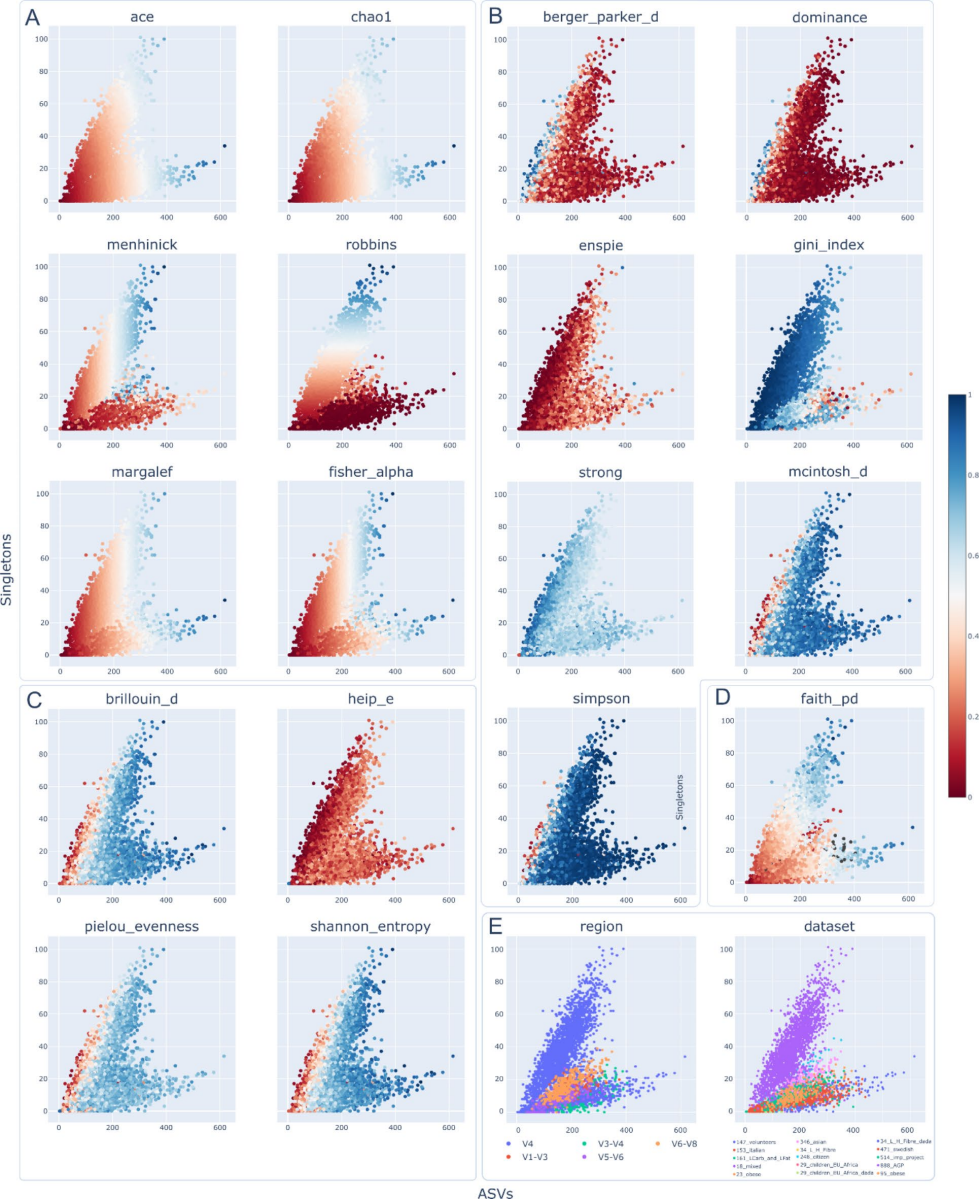
Relationship between singletons (Y axis) and the number of ASVs (X axis) of the 4,596 samples. All plots display the same data points, but each plot was colored according to a different alpha diversity metric. Colors on plots A, B, C and D correspond to a normalized value (proportion) of the corresponding alpha metric. (**A**) The scatter plot of the metrics grouped in the Richness category (minus the Observed metric, which are the values on the X axis), (**B**)Dominance metrics, (**C**) shows Information metrics and (**D**) Faith metric (phylogenetics). (**E**) Depicts samples colored according to the 16 S amplified region and the original study.

Throughout this study, we identified two key factors relevant to the estimation of different alpha diversity metrics, as discussed in the Methods section (Theoretical Analysis): The total number of Amplicon Sequence Variants (ASVs), and ASVs with only one read (singletons). It was verified that sequencing depth had no impact on the total number of ASVs and singletons (Supplementary Information, Figure S2, Table S3). Given this, alpha diversity metrics were calculated with non-rarefied data to preserve as much information as possible when calculating them. All results were also validated using rarefied datasets. As described in Methods, all samples were processed using DADA2 and DEBLUR. It is important to note that DADA2 removes all singletons from the samples as part of its denoise algorithm. Since the number of singletons is needed to calculate some of the alpha diversity metrics, all following analyses were performed with the data produced with DEBLUR. Figure 1 shows all samples according to their observed features (total number of observed ASVs) and singletons values. Figure 1A shows that values from metrics included in the Richness category depend on these two key factors. The color scale for each graph depicts the normalized value of each metric (min-max normalization). All Richness metrics increase as there are more observed ASVs, except Robbins which depends on the number of identified singletons. Figure 1B shows Dominance metrics values according to the corresponding factors. In this category, analysis is more complex and requires detailed considerations. Berger-Parker and ENSPIE values tend to decrease when the number of ASVs increases. Due to the Simpson calculation formula, the previous trend is also present but with the opposite sign (dominance values close to the origin tend to decrease). Dominance values tend to decrease when singletons increase and the number of ASVs is less than 200 (for Strong and Simpson metrics, the opposite sign should be applied due to formula calculation). Figure 1C contains the same analysis applied to metrics in the Information category. Since all information metrics are constructed using the Shannon formula, they are expected to exhibit similar behavior, as observed. Conversely, in the Shannon, Pielou, and Brillouin plots, the behavior identified in the dominance category is observed in samples with fewer than 60 singletons and fewer than 200 ASVs, but reversed, with values tending to decrease. Figure 1D shows Faith values according to the two factors, indicating that this metric depends independently on both factors. Samples with low Faith values that do not follow previous trends (located in the center of the plot) are isolated to the 248_citizen project. Figure 1E displays all samples colored by the 16 S amplicon used and their original dataset. A Kruskal-Wallis test verified that different 16 S amplicons significantly impact the number of observed features and measured singletons (Supplemental Figure S3, Supplemental Table S4).

Within each proposed category, Pearson’s linear correlation coefficient and Spearman ‘s rank correlation coefficients were calculated to identify correlations among metrics. Detailed scatter matrices showing the correlations between each category are included in the online Supplementary information (Supplementary Figures S1a to S1c). In the richness category (S1a), Chao1 and ACE show the strongest linear correlation between them, while Margalef and Robbins show some variation, but are still highly correlated to Chao and ACE. Menhinick presents a slope variation that is dataset dependent. Robbins, as shown before, is calculated based on the total number of singletons instead of number of ASVs, and no strong correlation is found. All richness metrics except Robbins are highly correlated with each other and the number of ASVs, implying that differences in their formula have no relevant impact when applied to microbiome data.

**Fig. 2:**
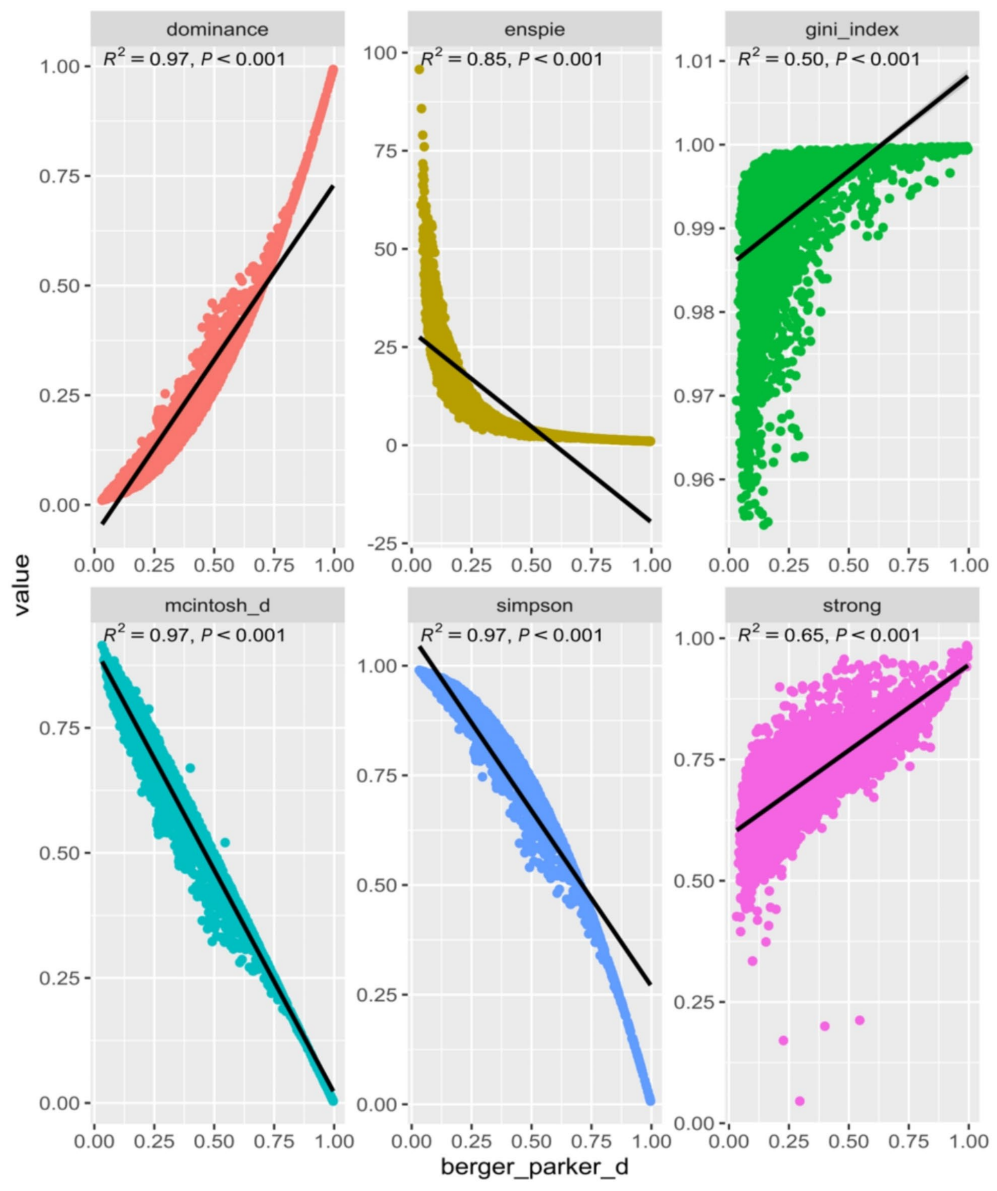
Scatter plot of each dominance metric between the corresponding value of the metric (Y axis) and Berger-Parker value (X axis) of the 4,596 samples.

Strong nonlinear correlations can also be observed between dominance metrics (S1b). Dominance and ENSPIE are variations of the Simpson metric, and are strongly correlated with each other as expected. McIntosh and Berger-Parker are also correlated to these metrics. It is of particular interest that Berger-Parker has a clearer biological interpretation from its mathematical formula (the proportion of the most abundant taxa). Figure 2 presents, for each metric in the Dominance category, a linear regression using all samples, with the X-axis representing the Berger-Parker value and the Y-axis representing the corresponding metric value. The McIntosh and Simpson models show a good fit. In the case of ENSPIE, an exponential transformation fits better than a linear model. The Gini index does not fit well in this model, nor does it fit well when a correlation model is applied to all dominance metrics.

**Fig. 3:**
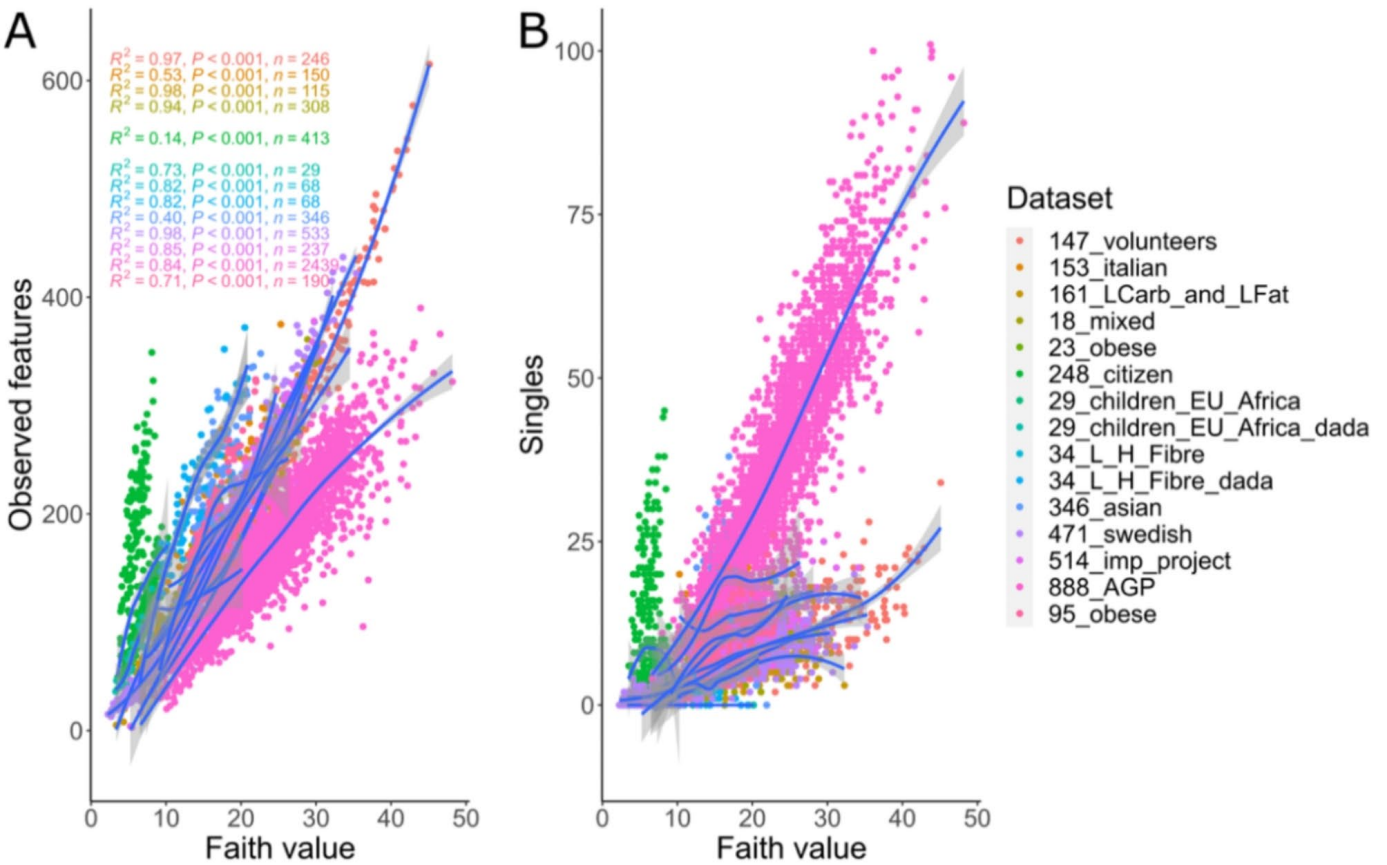
Scatter plot and local polynomial regression fitting between Faith metric (X axis) versus (**A**) Observed features and (**B**) singletons (Y axis) for each dataset. Determination coefficient(R^2^) is shown.

Faith is the phylogenetic metric most applied in experimental studies (See Supplementary table S1). Observed features and singletons are determinant factors, as shown in Fig. 1D. Based on this analysis, a local polynomial regression fitting was applied on the relationship between *observed features* (the selected metric proposed as representative for the Richness category in the Discussion section) and Faith, for each dataset. Figure 3A shows that the calculated regression models are close to a polynomial regression, and that the determination coefficients (R^2^) are statistically significant except for three models that are not well fitted due to the nature of its samples and, more specifically, because the number of Observed features is low (for example 248_citizen, green dots). A polynomial regression was also performed for singletons and Faith values, and it is shown in Fig. 3B. No correlation was found between Faith and dominance metrics (data not shown).

A similar behavior can be observed between metrics in the Information category (S1c) where all metrics show strong correlation between them. This is expected as all information metrics use Shannon’s entropy as a reference in their mathematical formulation, and its implications will be further discussed in the Discussion section.

After carrying out empirical experiments on publicly available human microbiome data, we validated our findings by applying the alpha diversity metrics to 7 synthetics datasets (See Methods, Supplementary Figure S6 and S7). For most metrics their value range changed depending on the distribution, but after performing a min-max normalization to analyze their behavior, we did not see meaningful differences related to the aim of this work between distributions. Following our previous findings, most abundance metrics increase according to variations on the total number of ASVs, except for Robbins that is influenced by the presence or absence of singletons within the sample. Dominance metrics were particularly affected by the artificial unevenness ratios (2x, 10x and 100x). Finally, information metrics increased when ASVs also increased, and decreased when there is an imbalance in the microbiome.

### Practical recommendations taken from observations and theoretical analysis

From the theoretical analysis conducted in the Methods section (see Theoretical Analysis subsection), we identified four categories that group the metrics according to the aspect of alpha diversity they focus on. Within each category, the metrics share similar objectives and related formulas. While considering all metrics results in significant diversity in their formulations, this complexity makes the analysis challenging. However, our analyses revealed that many of these metrics are highly correlated with each other, indicating that the complexity in the formulations is not reflected in the achieved results. Therefore, we recommend using the simplest metric within each category that demonstrates behavior similar to that of the more complex counterparts.

Taking this into account, along with the observations described above and the theoretical analysis of each metric’s mathematical formulation, we have developed a set of final practical recommendations to be considered for future studies:

- Report the bioinformatics tool used to perform the denoise process and inform the singleton values to better evaluate richness and dominance metrics.
- Provide at least one metric from each of the following complementary alpha diversity categories: Richness, Dominance, Information and Phylogenetics. The recommended eligible metrics are mentioned in the following bullet points.
- For the *Richness* category one of the following metrics should be informed: Observed, ACE, Chao1, Fisher, Margalef, McIntosh and Menhinick. Any of the previous metrics could be used because they are all correlated; however, we recommend reporting the number of observed features given the simplicity of its calculation technique and its intuitive biological interpretation. We also propose the inclusion of the Robbins value (the probability of not observing present taxa in the sample).
- For the Dominance category, we recommend using the Berger-Parker metric, which indicates the proportion of the most abundant taxa relative to all others. When high dominance is observed in a sample, it is advisable to identify the most predominant microbe. If the predominant microbe can be identified at the species taxonomic rank, or even at the strain level, it is recommended to report its functional annotations as a preliminary step for potential further functional analysis. These annotations could include GO terms, Clusters of Orthologous Genes (COGs) abundances, possible KEGG annotations, or similar functional data. However, if it is not possible to identify the microbe at the species level and only a higher taxonomic rank is identified, functional annotations from amplicon sequencing are not recommended. This is because their robust resolution is still debated and may not provide reliable insights.
- For the *Phylogenetic* category, report the Faith value.
- Report the Shannon value to inform not only the entropy but also as a parameter that summarizes/weighs both richness and dominance categories in one value (see Discussion section).
- Include a biological interpretation and conclusions for each reported metric considering the microbiome’s aspect it measures, as well as an interpretation of all metrics collectively.
- Alpha diversity metrics for samples processed under different 16 S amplicons should not be compared, as this was proven to affect the number of observed features and singletons observed, and consequently, the calculated values of the metrics.

## Discussion

The processing of microbiome sequencing raw data presents numerous bioinformatic challenges to achieve unbiased results, including tasks such as merging and removing primers and barcodes, denoising, dereplication, chimera removal, and taxonomic classification, among others. Moreover, the study of microbiota diversity encompasses diverse and distinct concepts that should be separately considered. For instance, within alpha diversity, metrics like dominance and richness capture distinct aspects of community composition and structure. Meanwhile, within beta diversity, methodologies such as distance calculation methods and dimension reduction techniques are crucial for understanding compositional differences between microbial communities. For this purpose, metrics used to characterize these concepts and the tools that enable their estimation have been adapted from other disciplines, such as the study of animal and plant communities, economics, sociology, or mining^17–19^, and are commonly applied without major distinctions^20–22^. However, microbiota data present specific characteristics that necessitate special attention. These include issues such as compositionality, sparsity, the double zero problem, overdispersion, zero-inflated problems, and sampling bias. These unique features require tailored approaches and methodologies to ensure accurate and meaningful analysis of microbiota diversity and composition.

Based on this, our focus has been on the fundamentals, assumptions, and formula calculations for selected metrics. Some metrics were excluded because they were not used by the scientific community on recent microbiota studies (Esty, Good’s coverage of counts) or their mathematical formula is complex, derived from a previous one, with its underlying concept close to another with a simpler formula (Renyi entropy, Tails, Kempton Taylor’s Q, Lladser’s point estimate, Rao, InvSimpson) or their fundamentals and assumptions are not directly applicable to microbiota data (Hill, Esty). Metrics selected were primarily focused on one alpha diversity aspect, ensuring consistency in assumptions and formulas. Under these considerations, we identified four distinct categories that complement each other and collectively provide a comprehensive overview of alpha diversity. The following paragraphs include a brief explanation of each proposed category along with the recommended metrics:

### Richness

 The number of different microorganisms in a sample. In experimental studies, instead of the number of *observed* microorganisms (detected by the sequencing technique) it is more relevant to estimate the number of *present* microorganisms in the population. The number of microorganisms not observed but present in a sample is usually estimated through singletons reads, i.e., a sequence detected only once within the sample. From a bioinformatic point of view, these reads can be treated as sequencing error candidates or, conversely, relevant information to infer low-abundance microorganisms that are present in the sample but not sequenced. Under this assumption, some alpha diversity metrics (Chao1, for example) attempt to estimate the real richness from the low-abundance information. In this way, the bioinformatic analysis of singletons is also a challenge because tools that perform the denoise process have different algorithmic approaches. DADA2^23^ and DEBLUR^24^ not only have different algorithmic denoising techniques but also have relevant differences in their singletons treatment approach. DADA2 removes singletons and DEBLUR keeps them. This difference should be considered as a source of variability when reporting the estimated microorganism richness of the sample, if the denoise tool is not reported. This recommendation is also significant since the number of identified taxa is tool dependent and therefore alpha diversity metrics can be biased^25^. In the case of using DADA2, Chao1 and Robbins should not be used because they require singletons for its calculation.

### Dominance

The degree of balance (or evenness) observed in the richness distribution of microorganisms in a sample. The goal of this category of metrics is to inform if some microorganisms are dominant over others in terms of richness. After observing each formula calculation of the metrics grouped in the dominance category and the result of applying them to the 4,596 samples, it was found that Berger-Parker (the metric that reflects the ratio between the most dominant microorganism and all other microorganisms in the sample) best represents this category. As it is correlated with the proportion between the most dominant over the second most dominant microorganism (see online Supplementary Information, Figure S5), it could be considered that the two most dominant microorganisms are determinant factors of dominance metric value, no matter the selected metric to inform about this category. One identified benefit of providing Berger-Parker as a reference metric of Dominance category is that the biological meaning of the reported metric is clearer and easy to inform, namely the proportion of the most dominant microbe over the total number of microbes present in the sample.

### Phylogenetics

 It is a measure of biodiversity that informs the phylogenetic distance between microbes within a corresponding sample. This category, unlike the other ones, is not inherited from other disciplines. Faith is the only Phylogenetic metric broadly adopted in experimental studies, and its value is strongly linked to the number of observed features and singletons.

### Information

 It is a measure inherited from the physics discipline thought to inform the entropy of a system. Shannon is the most used in experimental studies and, in fact, other metrics included in this category (Brilloun, Heip y Pielou) use this metric as a reference. In microbiome studies the Shannon index is supposed to reflect how many different microbes are in the sample and how evenly they are distributed within a sample: it is a number that aims to encompass and integrate richness and dominance information. The more microbes in a sample are, the higher the value for the Shannon index is; and the less the inequality of relative abundances is, the higher the Shannon index is too. Since the three proposed metrics as representative for the categories Information (Shannon), Richness (Observed_features) and Dominance (Berger-Parker) are correlated (Supplementary information, Figure S5) we state that, if richness and dominance metrics are reported, then Shannon does not provide new information to the alpha diversity big picture. Furthermore, the Shannon value is elusive to interpret from a biological point of view. On the other hand, if Shannon is the only reported metric in a given study, it could also be assumed that it provides a weighted/synthesized representation of information and richness categories, in just one value. Thereby, the complex nature of the biological interpretation of this metric remains an issue.

**Fig. 4:**
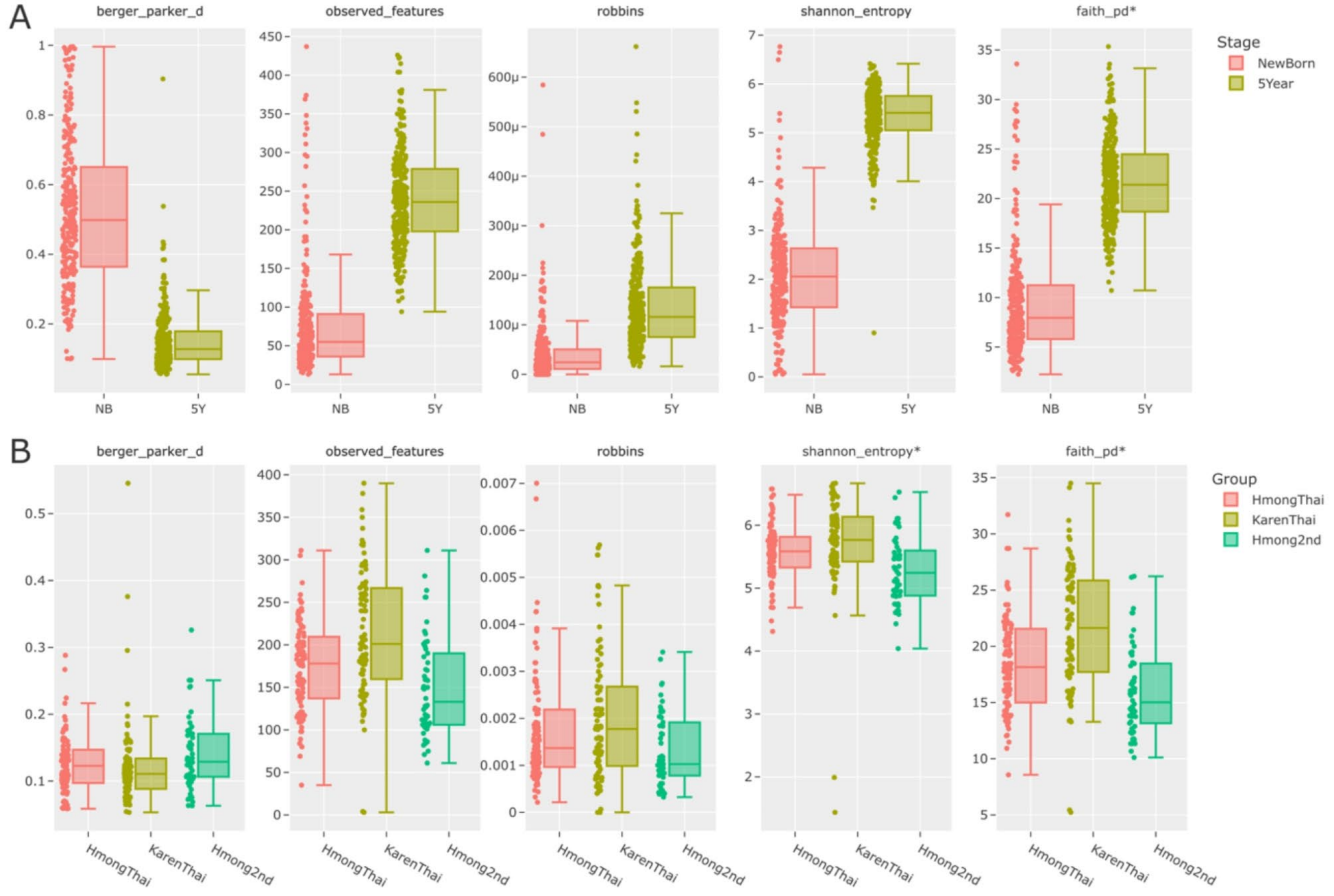
Boxplots of each recommended alpha diversity metric for two datasets analyzed in this article, 471_swedish (**A**) and 514_imp_project (**B**). Metrics with an asterisk (*) were reported by authors in their original studies.

**Practical examples and applications.** Taking into consideration the previously proposed metrics under their respective categories, practical examples of how these recommendations can enhance studies are as follows. Three examples are illustrated and described (Fig. 4), utilizing data from two analyzed studies (projects 471_swedish and 514_imp_project, according to Table S3 codes). These practical examples aim to demonstrate how implementing these recommendations in result analysis enhances the quality and comprehensiveness of information, facilitating improved interpretation when working with microbiome data.

#### Example 1

In this first case (project 471_swedish), the dominance (or evenness) category was not computed. The authors reported an increase in alpha diversity (specifically reported as richness, computed using phylogenetic diversity) as children grew older, which is accurate. However, the dominance category offers additional insights into this observation, demonstrating that as richness increases, dominance decreases (Fig. 4.A). Considering this provides complementary information that can illuminate aspects not previously addressed, as these were obscured by metrics that do not incorporate this information. In essence, as individuals in the trial age, both the number and diversity of observed microbes increase, as measured by the phylogenetic diversity metric (and also by observed features). Furthermore, this biological interpretation introduces a new perspective: with increasing age, there is a reduced dominance of previously predominant microbes identified in newborn cases, possibly reflecting microbial community colonization and stabilization over time.

#### Example 2

In the second case (project 514_imp_project), among the five proposed metrics, the authors reported Shannon and Faith, which showed the highest variance. However, upon analyzing the total observed features, singletons (using Robbins), and evenness in dominance, no significant differences were observed (Fig. 4.B). In this context, emphasizing Faith may have a greater impact, suggesting that any observed differences could be attributed more to phylogenetic origins and/or adaptations. Considering this, only a comprehensive comparison of all five metrics enables us to hypothesize about phylogenetic origins/adaptations, as other aspects showed no substantial differences. Standardizing alpha diversity reporting according to these recommended metrics will facilitate a more comprehensive description of such scenarios, utilizing all available information effectively.

#### Example 3

Finally, the third example is drawn from the same project 514_imp_project, where no dominance metrics were computed. According to Robbins (Fig. 4.B), there appears to be a subsampling artifact in the KarenThai cohort, indicated by a higher mean estimate of unobserved microbes compared to the others. This suggests that alpha diversity metrics might exhibit different trends if the KarenThai cohort were more representative of its true diversity. This hypothesis, if validated, could yield different results for this cohort compared to others, influencing biological interpretations due to potential subsampling issues. Essentially, the diversity observed in the KarenThai cohort may differ from what is reported. To verify this, a new sampling approach should encompass a broader range of microbiome patterns, involving a larger sample size from the trial.

These recommendations are applicable to both existing and future research studies, enhancing the standardization, consistency, and robustness of analyses conducted. This approach aims to improve the capture of biological diversity and, consequently, enhance interpretations of microbiome data.

## Methods

### Selection of alpha diversity metrics

Alpha diversity metrics included in this study were selected from a detailed examination of 68 microbiome studies (see online Supplementary Information, Table S1) conducted on human or animal samples, or that are included in the bioinformatics suite QIIME 2.

68 studies were selected to identify the most applied alpha diversity metrics in research articles. Scientific projects published after 2008 that performed the sequencing of the 16 S ribosomal biomarker were included. The most recent studies (10 from 2019, 12 from 2020 to 3 from 2021) were prioritized, although three older ones (2008, 2010 and 2012) were also included due to their experimental design quality and sample size. These studies perform at least one of the following alpha diversity metrics: Chao1, Shannon, richness, Berger-Parker, ACE, Simpson, Faith and Jost. All these metrics are included in this work except for the Jost metric, designed for ecological communities. Its main assumption is based on the formula $\:\alpha\:+\beta\:=\gamma\:$ (where $\:\alpha\:$ is alpha diversity, $\:\beta\:$ is beta diversity and $\:\gamma\:$ is gamma diversity) and as $\:\gamma\:$ -the total species diversity in a landscape- is not used in microbiome diversity, it was considered that Jost metric should not be included in the selected metrics.

In addition, alpha metrics implemented in the QIIME2^26^ suite -some of which were not used in the 68 studies mentioned above- were also included because it is one of the most widely used packages for bioinformatic analysis of microbiota studies^27^.

### Theoretical analysis

The theoretical analysis performed on each metric was carried out by considering which aspect of biodiversity is measured, which scientific discipline the metric was derived from, how often it is applied in the microbiota field, the variables that appreciably affect/impact the proposed metric and whether the metric includes an estimation or is reflecting observed data, i.e. if the metric is generating a new concept (like information theory, entropy, evenness) or is it just an alias for a quantitative value that is intrinsic to the dataset (number of ASVs, singletons)^28^. Synonymous metrics with significant similarity in the mathematical formula or correlated metrics were grouped together and the chosen one was selected because of its easy calculation method or the biological interpretation of the result. The 19 selected metrics were grouped into four broad categories, i.e., richness-, dominance-, phylogeny-, or information-based. Below we provide a summary of the theoretical analysis of each selected alpha metric, grouped into these four categories (see Table 1 for the corresponding mathematical formulas, together with their key involved parameters).

### Richness

These metrics report on the number of species in a sample. Since species definition in microbes is less defined than in e.g., plant or insect studies, and since microbiome studies usually are based on genetic analysis, other methods of grouping can be used instead of “species”. Most commonly, highly similar microbial sequences are grouped into “OTUs” (operational taxonomic units) or “ASVs” (amplicon sequence variants), which roughly equate species or strains, depending on which sequence identity cutoffs are used. Here, we will use the more general term “taxa”, to indicate the microbial equivalent of “species”.

- **Chao1**^29^: Point estimator based on observed richness with an added term that infers the number of taxa not observed -due to sequence technique constraints- but present in the sample according to a rate between the number of singletons and the number of doubletons.
- **ACE** (Abundance-based Coverage Estimator): Point estimator that follows the same strategy of Chao1: a baseline given by the observed abundance and an addition aimed to infer the microorganisms not observed but present in the sample. The difference relies on the fact that ACE applies a more sophisticated statistical technique for the inference. This proposal includes a threshold to cluster “rare” taxa (not abundant) from “not rare” ones. In the empirical values evaluation of this work Chao1 and ACE have similar results.
- **Fisher**^30^: A univariate parametric estimator ($\:\alpha\:$ value) that assume that abundance values follow a logarithmic series model. It was widely used in entomological research. The parameter $\:\alpha\:$ has no biological interpretation. This metric comes from other disciplines and therefore its parametric proposal does not fit very well to the nature of the samples.
- **Margalef**^31^: This metric proposes to correct biases caused by variations in sampling from the total number of reads.
- **Menhinick**^32^: This metric follows the same proposal of Margalef metric, but it differs only in the way to transform the total number of reads. Margalef reports a lower richness over Menhinick in samples with a large number of microorganisms. An identified constraint of both metrics (Margalef and Menhinick) comes from the fact that they use the total number of reads to fit the reported bias. In this way they do not discriminate between the number of microorganisms (abundance / richness) and how they are distributed (dominance).
- **Observed**: The number of observed features present. Features may refer to Amplicon Sequence Variant (ASVs) or Operational Taxonomic Units (OTU) depending on the denoising tool.
- **Robbins**: Is a proportion between the number of singletons and the total number of ASVs. In this way, we consider that this metric should not be considered as an alpha metric diversity but a likelihood of the existence of present and not observed taxa in the sample.

### Dominance

- **Berger-Parker**^33^: This metric is a ratio between the abundance of the most abundant ASV and the number of ASVs in the sample. In this way, it informs the distance of a given ASV from the most abundant ASV. Compared with other dominance formula metrics, it is easy to calculate and has a clear biological interpretation.
- **Simpson**^34^: Measures the probability that two microorganisms randomly selected from a sample belong to the same species. It is usually used in ecology research. In this way, 0 represents infinite diversity and 1, no diversity. That is, the bigger the Simpson metric, the lower the diversity. As this is unintuitive the value is usually subtracted from 1. Another option is to obtain the inverse value (1/Simpson). In both cases the resulting value does not match with a biological interpretation.
- **Dominance**: As reported by the QIIME2 package and is often used in different studies. Its formula is 1-Simpson.
- **ENSPIE** (Effective Number of Species, Probability of Interspecific Encounter): This metric is equivalent to the inverse of the Simpson metric.
- **Gini**^35^: This metric measures the inequality among values of a frequency distribution. It is usually applied in economics and, from a conceptual point of view, is the area difference between the cumulative distribution of a perfect evenness richness and the real distribution curve of the sample, following the Lorenz model. It ranges between 0 and 1 where 0 corresponds to a maximum degree of homogeneity and 1 indicates the maximum degree of inequality. After a detailed study and a simulation (not shown in this work) it was found that assumptions and required parameter settings do not fit well to microbiome data. It is coherent with the empirical study results of this work where it was found that mean Gini value 0.99 and its standard deviation 0.0083.
- **McIntosh**^13^: This metric expresses the heterogeneity of a sample in geometric terms. Formula requires the use of a variable (*U*) that contains the distance of the sample from the origin in an S dimensional hypervolume. There is no easily understandable biological interpretation related to this variable. It has been little used in literature.
- **Strong**: This metric has a similar Gini conceptual proposal, and they share the same conclusions and remarks.

### Phylogenetic

- **Faith**^36^: Measures the amount of the phylogenetic tree covered by the community. Its calculation is performed by an algorithm on a tree, not from a mathematical formula. The value is the sum of the branch lengths of a phylogenetic tree connecting all species in the target assemblage. In this way, a higher value means more branches, means more richness. This metric does not consider species abundances and depends on the sequencing depth, denoising method, rarefaction depth and phylogenetic tree^37,38^.

### Information

- **Shannon**^39^: This metric measures uncertainty about the identity of species in the sample, and its units quantify information. It comes from Information Theory where it quantifies the entropy of a system, and it has also been a popular diversity index in ecological literature. This metric depends on the sample size and intends to provide a weighted average, or a synthesis, between the richness and the dominance of a sample. Other information metrics derive from this formula. The main hurdle could be found in the interpretation of the value since it is not possible to know if a given variance is due to richness in the sample, or its dominance or both.
- **Brillouin**^40^: This metric has a similar theoretical proposal and results than Shannon metric, but it assumes that there is no uncertainty in the population from where sampling is applied. It has its origins in ecology and is useful when the entire population is known or when the randomness of the sample cannot be guaranteed. This metric is not applicable to sequencing data because the completeness of the census cannot be guaranteed. It is difficult to calculate, and its correct biological interpretation even more difficult.
- **Heip**^41^: This proposal differs on the Shannon index since it does not depend on the sample size: it is a proportion of entropy.
- **Pielou**^42^: This proposal is mainly similar to Heip but they differ in the normalization strategy. While Heip divides the entropy by the total number of taxa, Pielou divides the log of the total number of taxa.

**Table 1:** Mathematical formulas used in the alpha diversity metrics included in this study, grouped by suggested categories.

Category	Name	Expression	Parameters
Richness	Chao1	$\:\:S\:+\:\frac{{a}^{2}}{2b}$	*S*: number of observed taxa (e.g., species, OTUs, ASVs, sequence variants). *a*: number of singletons (taxa observed only once) *b*: number of doubletons (taxa observed two times).
	ACE	$\:\widehat{{S}_{abun}}+\frac{\widehat{{S}_{rare}}}{{\widehat{C}}_{rare}}+\gamma\:\left(CV\right)\:$	$\:{S}_{abund}={\sum\:}_{i>k}^{k}{f}_{i}$ is the sum of abundances of the taxa more abundant by considering k the threshold from where taxa are considered “abundant.” $\:{S}_{rare}={\sum\:}_{1}^{k}{f}_{i}$ is the sum of abundances of the taxa considered “not abundant.” $\:{n}_{rare}={\sum\:}_{1}^{k}{f}_{i}$ is the sum of abundances of the taxa considered “abundant.” $\:{\widehat{C}}_{rare}=1-\frac{{f}_{1}}{{n}_{rare}}$ is the size of the group that contains “not abundants” taxa. $\:\gamma\:\left(CV\right)$ is an estimation function that fits the heterogeneity in the “not abundant” taxa.
	Fisher	$\alpha x~,~\frac{{\alpha x^{2} }}{2},\frac{{\alpha x^{3} }}{3},~\frac{{\alpha x^{i} }}{i},...,\frac{{\alpha x^{n} }}{n}$	x: abundance of ASV i. $\:\alpha\:$: is the parameter of the metric.
	Margalef	$\:\frac{(S-1)}{Ln\:N}$	*S*: number of observed taxa (e.g. species, OTUs, ASVs, sequence variants). N: total number of reads.
	Menhinick	$\:\frac{S}{\sqrt{N}}$	*S*: number of observed taxa (e.g. species, OTUs, ASVs, sequence variants). N: total number of reads.
	Robbins	$\:\frac{a}{S\:+\:1}$	a: number of singletons. *S*: number of observed taxa (e.g. species, OTUs, ASVs, sequence variants).
Dominance	Berger-Parker	$\:\frac{{S}_{max}}{S}\:$	$\:{S}_{max}:$ number of observed taxa in the most abundant taxon. *S*: number of observed taxa (e.g. species, OTUs, ASVs, sequence variants).
	Simpson	$\:1-{\sum\:}_{i\:=\:1}^{S}{p}_{i}^{2}\:$	$\:{p}_{i}$: the proportion of the *i* ASV.
	ENSPIE	$\:\frac{1}{{\sum\:}_{i=1}^{s}{p}_{i}^{2}}$	S: number of ASVs. $\:{p}_{i}$: the proportion of the *i* ASV.
	Gini	$\:\frac{2}{m{S}^{2}}\left(\sum\:_{i=1}^{n}\left(S+1-i\right){x}_{i}\right)-\frac{1}{S}$	*S*: number of observed taxa (e.g., species, OTUs, ASVs, sequence variants). $\:{x}_{i}:$ abundance of the *i* taxa ordered decreasingly. $\:m$: x mean.
	McIntosh	$\:\frac{S\:-\:U}{S\:-\:\sqrt{S}}$	*S*: number of observed taxa (e.g., species, OTUs, ASVs, sequence variants). $\:U\:=\:\sqrt{{\sum\:}_{i\:=\:1}^{S}{n}_{i}^{2}}$ where $\:{n}_{i}\:$is the number of microorganisms of the *i* taxa.
	Strong	$\:{{max}}_{i}\left[\left(\frac{{b}_{i}}{S}\right)-\:\frac{i}{R}\right]\:$	$\:{b}_{i}$: the cumulative abundance til taxa *i* ordered decreasingly. *S*: number of observed taxa (e.g., species, OTUs, ASVs, sequence variants). R: The number of taxa. $\:ma{x}_{i}$: the max of all *i*.
Information	Shannon	$\:-{\sum\:}_{i\:=\:1}^{S}p\left({x}_{i}\right)lo{g}_{2}\left(p\right({x}_{i}\left)\right)\:$	$\:{p(x}_{i})$: the likelihood to observe the *i* taxa. Namely the rate between the number of reads for taxa *i* and the total number of reads. *S*: number of observed taxa (e.g., species, OTUs, ASVs, sequence variants).
	Brillouin	$\:\frac{\text{ln}S!\:\:-{\sum\:}^{\:}\text{ln}{S}_{i}\:!}{S}$	*S*: number of observed taxa (e.g., species, OTUs, ASVs, sequence variants).
	Heip	$\:\frac{\left({e}^{H}\:-\:1\right)}{\left(S\:-\:1\right)}$	$\:H$: Shannon entropy. *S*: number of observed taxa (e.g., species, OTUs, ASVs, sequence variants)
	Pielou	$\:\frac{H}{\text{ln}\left(S\right)\:}$	$\:H:$ Shannon entropy. *S*: number of observed taxa (e.g., species, OTUs, ASVs, sequence variants)

A formula for the Faith metric, under the Phylogenetic category, was not included because the metric does not perform a mathematical approach but an algorithmic one, which is continuous suffering changes, but basically it is computed by summing the branch lengths (edge weights) of the phylogenetic tree that exclusively represents the sequences contained in a biological sample.

### Selection of sequence datasets for metric testing

The 19 selected metrics were applied to publicly accessible sequence data from 16 S ribosomal biomarker sequencing files, obtained from a total of 4,596 stool samples described in 13 publicly available human microbiome studies published from 2008 to the present (see online Supplementary Information, Table S2). Projects were excluded if they lacked associated metadata, but not if there were missing samples reported in the metadata. The term “reference cohorts” is used to describe what are usually called “healthy participants“, which we find to be imprecise due to the complex nature of microbial communities and their interactions with the host. Therefore, it is more accurate to refer to these groups as reference cohorts or reference control groups^43,44^. Since alpha diversity is a metric applied within each sample, conditions/groups are not relevant for this work. However, only reference cohorts were considered to avoid potential bias from lower observed diversity, as it is well known that diversity is a common indicator of gut health, which is generally lower under disease conditions. Raw data (sequencing files) and metadata from the selected studies were downloaded from the repositories of the National Center for Biotechnology Information^45^ (NCBI) or the European Molecular Biology Laboratory^46^ (EMBL).

### Bioinformatic processing

Downloaded samples were processed with the QIIME2 tool suite, installed on a conda environment on a personal computer with Ubuntu 21.04. Samples identified as paired-end that only had a single read were removed. After performing QC for each dataset independently (read quality, adapter trimming), bioinformatic processing of all projects was performed altogether in two pipelines (qiime2 and dada2). Parameters and databases for taxonomy classification were the same for all processed projects. Alpha diversity metrics were computed for each sample separately. Particularly, DEBLUR was used for noise removal, dereplication, chimera removal and ASV selection.

All datasets were also processed with DADA2 to verify its impact on singleton values, but all further tests were performed only on the DEBLUR results. Likewise, all datasets were rarefied to a suitable sequencing depth following standard procedures to validate our claims (data available in repository), but all tests were performed with non-rarefied data. Phylogenetic trees were created using the q2-phylogeny plugin. Selected alpha diversity metrics were calculated for each sample using q2-diversity (from qiime2 package), combined with the metadata, and aggregated for all datasets. For systematic alpha metrics generation on all studies, Bash and Python scripts were created. For all further visualizations and comparisons, Jupyter Notebooks^47^ were designed in the Visual Studio Code^48^ environment. Pandas^49^ and Numpy^50^ were used for managing data frames. Numpy^50^, Scipy^51^ and R^52^ were used for statistical analysis and regression models, whereas Plotly^53^ and Seaborn^54^ for visualizations.

### Synthethic datasets

We generated seven datasets, each with approximately 2,500 samples. Each sample included between 50 and 500 different ASVs (in steps of 10), 0 to 100 singletons (or 40% of the total number of ASVs in the sample, whichever came first, in steps of 3), and 40% of that number of doubletons. These values were selected based on careful analysis of experimental datasets. After determining the target number of singletons and doubletons, the remaining ASVs followed a normal, negative binomial, uniform, exponential, and Poisson distribution. Additionally, three more datasets were created using the negative binomial distribution as a base, where we controlled the ratio between the two most abundant ASVs (ASV2/ASV1). The negative binomial distribution had an average ratio of 0.8x, and we artificially created ratios of 2x, 10x, and 100x. All datasets were then imported into QIIME 2, and we recalculated the alpha diversity metrics for all, with the exception of ACE and Faith due to technical limitations.

## Electronic Supplementary Material

Below is the link to the electronic supplementary material.

Supplementary Material 1

## Data Availability

Project numbers (and doi) for sequencing data of the 13 projects used to perform the analyses are available online at the Supplementary Information, Table S2. Computer code and datasets generated/analyzed along the current study are available at https://github.com/MauroIb/alpha-diversities. The repository is also available with a permanent doi though Zenodo at https://zenodo.org/record/8170289. Complete QIIME artifacts are not included as the raw sequencing data belong to each of the dataset’s authors, but full references, SRA codes or similar, their ASV tables and the corresponding alpha diversity values are provided for each dataset within the repository.
