# Supplementary material for: Key features and guidelines for the application of microbial alpha diversity metrics
Source: Sci Rep. 2025 Jan 3;15:622. doi: 10.1038/s41598-024-77864-y (PMC11698868; doi:10.1038/s41598-024-77864-y)

Supplementary Information to:

**Key Features and Guidelines for the application of Microbial Alpha Diversity Metrics**

**List of Supplementary Information**

The following materials are included in the online Supporting Information, i.e., this pdf document.

**Supplementary figures to** “Results” and “Discussion” sections.

**Supp. Figure S1a.** Scatter matrix of the alpha diversity metrics included in the Richness category.

**Supp. Figure S1b.** Scatter matrix of the alpha diversity metrics included in the Dominance category.

**Supp. Figure S1c.**  Scatter matrix of the alpha diversity metrics included in the Information category.

**Supp. Table S1**. Experimental studies that include alpha metrics in its results.

**Supp. Table S2.** Metadata of the 15 public human microbiota experimental studies used to perform the selected alpha metrics.

**Supp. Figure S2**: Scatter plot of singletons and observed features (y axis) by sequencing depth (x axis).

**Supp. Table S3**: Spearman correlation coefficients calculated for observed features and singletons.

**Supp. Figure S3**: Boxplots for total observed features and singletons for each used 16S amplicon.

**Supp. Table S4**: Means and standard deviations for all 16S amplicon regions amplified in the used datasets.

**Supp. Figure S4.** Scatter plot that shows the relationship between Berger Parker metric and the proportion between the most dominant microbe and the second most dominant microbe, applied on the 4,596 samples.

**Supp. Figure S5.** Correlations among the three proposed metrics as representative for the categories Information (Shannon), Richness (Observed_features) and Dominance (Berger Parker).

**Supp. Figure S6**. Distribution of alpha diversity metrics when applied to synthetic datasets.

**Supp. Figure S7.** Relationship between singletons (Y axis) and the number of ASVs (X axis) of the samples in each of the synthetic datasets. Points represent samples, with each plot colored according to a normalized value (proportion) of the corresponding alpha diversity metric. Plots are organized with each metric in a different row and datasets in columns.

**Figure S1a.** Scatter matrix of the alpha diversity metrics included in the Richness category. The diagonal shows a histogram of each metric. The upper diagonal displays the linear correlation coefficients calculated using Pearson's method, with the R^2^ and p values for each correlation shown in red on the upper left side. The Spearman correlation coefficients (ρ\rho) and their corresponding p values are presented in blue at the upper right corner.


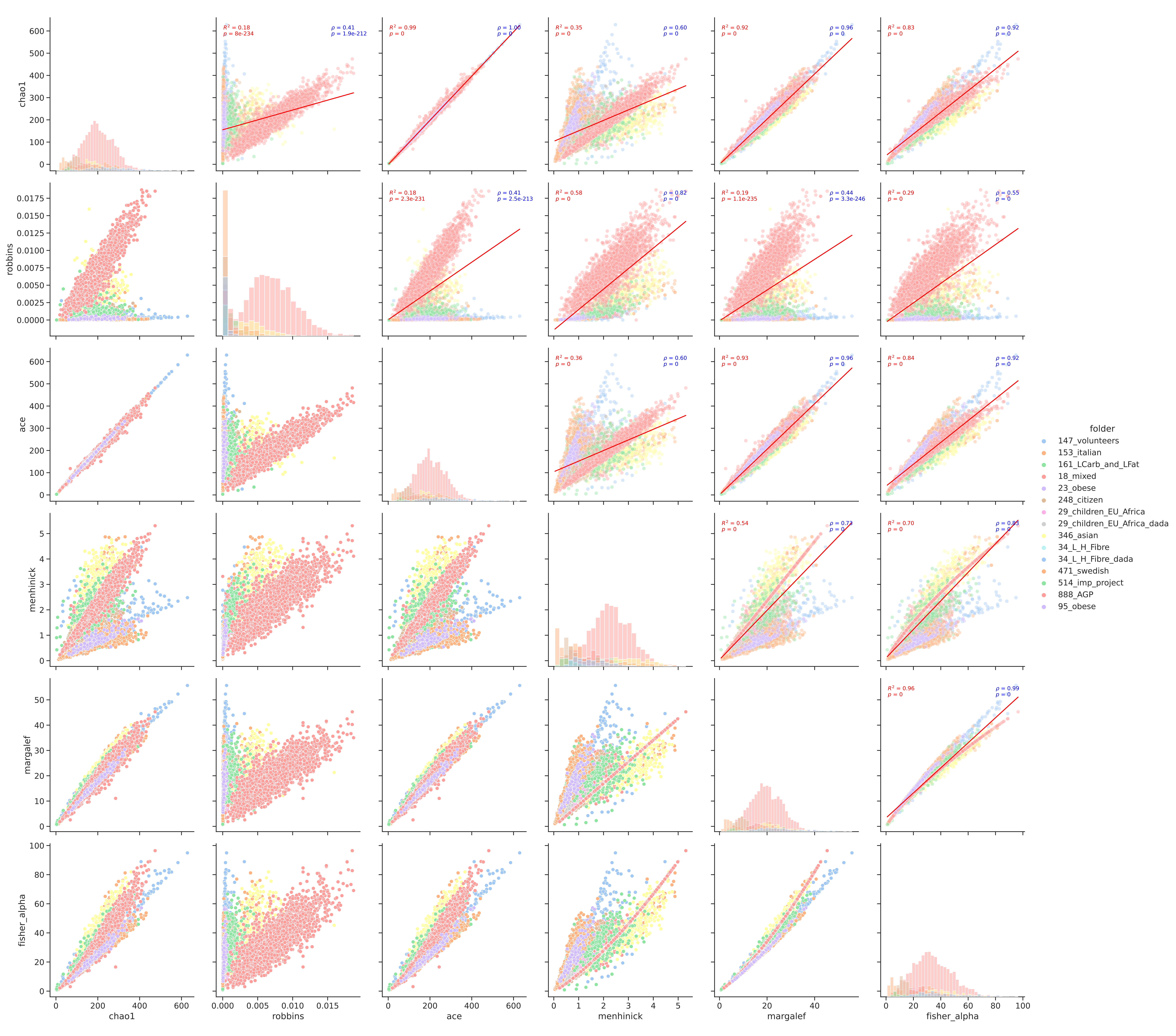


**Figure S1b** Scatter matrix of the alpha diversity metrics included in the Dominance category. The diagonal shows a histogram of each metric. The upper diagonal displays the linear correlation coefficients calculated using Pearson's method, with the R^2^ and p values for each correlation shown in red on the upper left side. The Spearman correlation coefficients (ρ\rho) and their corresponding p values are presented in blue at the upper right corner.





**Figure S1c.**  Scatter matrix of the alpha diversity metrics included in the Information category. The diagonal shows a histogram of each metric. The upper diagonal displays the linear correlation coefficients calculated using Pearson's method, with the R^2^ and p values for each correlation shown in red on the upper left side. The Spearman correlation coefficients (ρ\rho) and their corresponding p values are presented in blue at the upper right corner.


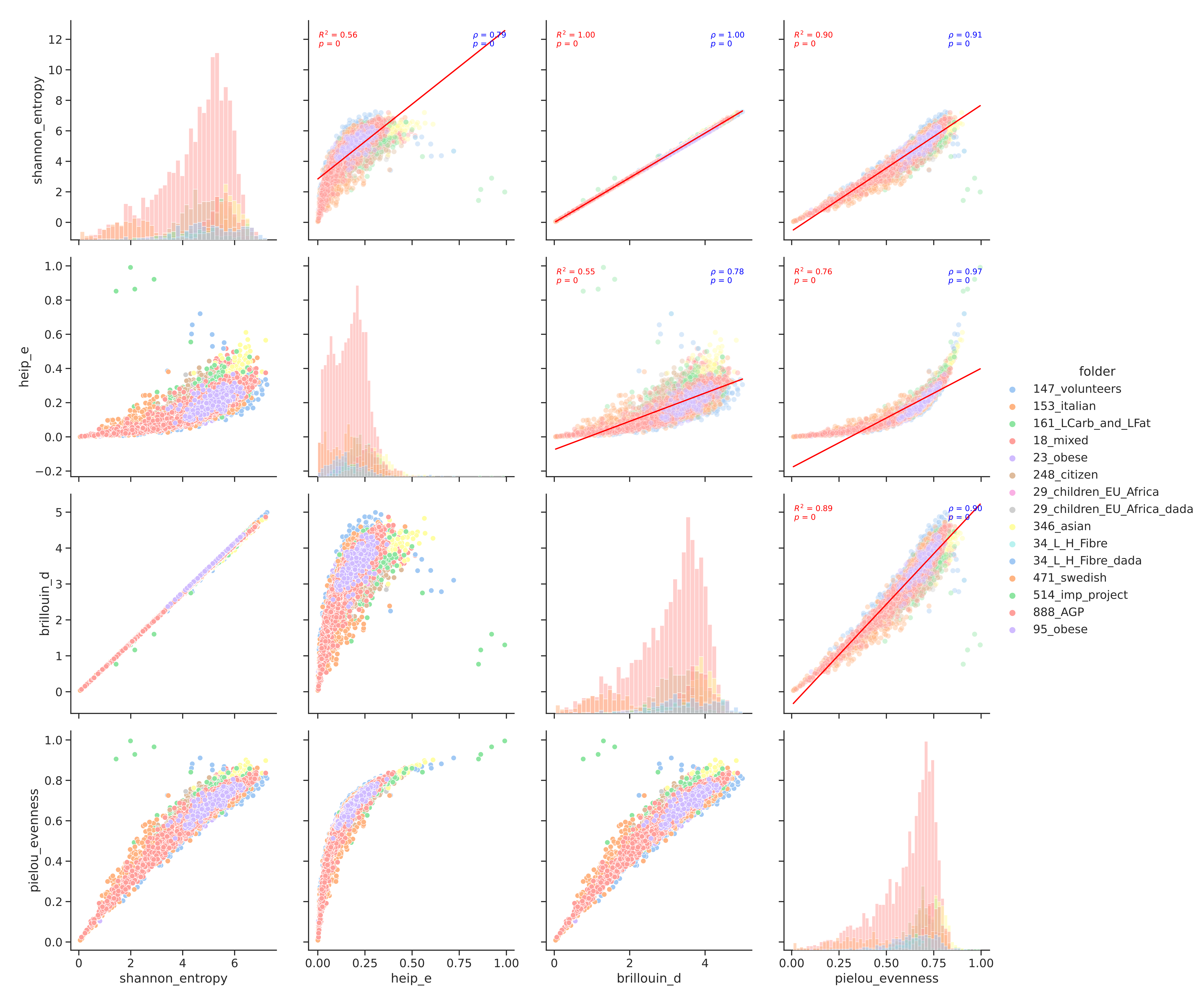


**Table S1.** Selected experimental studies that apply alpha-diversity metrics to its results.

| **Cod** | **Link** | **Chao1** | **Shannon** | **Observed** | **Simpson** | **Jost** | **Berger-Parker** | **ACE** | **Reyni** | **Faith** | **Fisher** | **Gini** | **Pielou** | **Magalef** |
| --- | --- | --- | --- | --- | --- | --- | --- | --- | --- | --- | --- | --- | --- | --- |
| **1** | [**Gut Microbiota and Dietary Intake of Normal-Weight and Overweight Filipino Children**](https://www.mdpi.com/2076-2607/8/7/1015) | **x** | **x** |  |  |  |  |  |  |  |  |  |  |  |
| **2** | [**Response of the Human Milk Microbiota to a Maternal Prebiotic Intervention Is Individual and Influenced by Maternal Age**](https://www.ncbi.nlm.nih.gov/pmc/articles/PMC7230887/) | **x** | **x** | **x** |  |  |  |  |  |  |  |  |  |  |
| **3** | [**Using compositional principal component analysis to describe children's gut microbiota in relation to diet and body composition**](https://academic.oup.com/ajcn/article-abstract/111/1/70/5621516?redirectedFrom=fulltext) |  | **x** |  |  |  |  |  |  |  |  |  |  |  |
| **4** | [**Structured exercise alters the gut microbiota in humans with overweight and obesity—A randomized controlled trial**](https://www.nature.com/articles/s41366-019-0440-y) |  | **x** | **x** |  |  |  |  |  |  |  |  |  |  |
| **5** | [**Longitudinal gut microbiome changes in alcohol use disorder are influenced by abstinence and drinking quantity**](https://www.tandfonline.com/doi/full/10.1080/19490976.2020.1758010) |  | **x** |  |  |  |  |  |  |  |  |  |  |  |
| **6** | [**Impact of Individual Traits, Saturated Fat, and Protein Source on the Gut Microbiome**](https://pubmed.ncbi.nlm.nih.gov/30538180/) |  | **x** |  |  |  |  |  |  |  |  |  |  |  |
| **7** | [**Randomized controlled trial on the impact of early-life intervention with bifidobacteria on the healthy infant fecal microbiota and metabolome**](https://academic.oup.com/ajcn/article/106/5/1274/4822328) |  |  | **x** |  | **x** |  |  |  |  |  |  |  |  |
| **8** | [**Multidomain analyses of a longitudinal human microbiome intestinal cleanout perturbation experiment**](https://journals.plos.org/ploscompbiol/article?id=10.1371/journal.pcbi.1005706) |  | **x** |  |  |  |  |  |  |  |  |  |  |  |
| **9** | [**Impact of Dietary Resistant Starch on the Human Gut Microbiome, Metaproteome, and Metabolome**](https://mbio.asm.org/content/8/5/e01343-17.short) |  | **x** |  |  |  |  |  |  |  |  |  |  |  |
| **10** | [**Exercise and associated dietary extremes impact on gut microbial diversity**](https://gut.bmj.com/content/63/12/1913.short) |  | **x** |  | **x** |  |  |  |  |  |  |  |  |  |
| **11** | [**Short-Term Effect of Antibiotics on Human Gut Microbiota**](https://journals.plos.org/plosone/article?id=10.1371/journal.pone.0095476) | **x** |  | **x** |  |  |  |  |  |  |  |  |  |  |
| **12** | [**Diet rapidly and reproducibly alters the human gut microbiome**](https://pubmed.ncbi.nlm.nih.gov/24336217/) |  | **x** |  |  |  |  |  |  |  |  |  |  |  |
| **13** | [**Alterations in intestinal microbiota of elderly Irish subjects post-antibiotic therapy**](https://academic.oup.com/jac/article/68/1/214/671295) | **x** | **x** | **x** | **x** |  |  |  |  | **x** |  |  |  |  |
| **14** | [**Differential Responses to Dietary Protein and Carbohydrate Ratio on Gut Microbiome in Obese vs. Lean Cats**](https://europepmc.org/article/MED/33178173) |  | **x** | **x** |  |  |  |  |  | **x** |  |  |  |  |
| **15** | [**Diabetic cats have decreased gut microbial diversity and a lack of butyrate producing bacteria**](https://pubmed.ncbi.nlm.nih.gov/30886210/) | **x** | **x** | **x** | **x** |  |  |  |  |  |  |  |  |  |
| **16** | [**Diabetic cats have decreased gut microbial diversity and a lack of butyrate producing bacteria**](https://pubmed.ncbi.nlm.nih.gov/30886210/) | **x** | **x** | **x** | **x** |  |  |  |  |  |  |  |  |  |
| **17** | [**Synbiotic-driven improvement of metabolic disturbances is associated with changes in the gut microbiome in diet-induced obese mice**](https://www.sciencedirect.com/science/article/pii/S2212877818312699#cebib0010) |  | **x** | **x** |  |  |  |  |  |  |  |  |  |  |
| **18** | [**Diet-Microbiome Interactions in Health Are Controlled by Intestinal Nitrogen Source Constraints**](https://www.cell.com/cell-metabolism/fulltext/S1550-4131(16)30553-8?_returnURL=https%3A%2F%2Flinkinghub.elsevier.com%2Fretrieve%2Fpii%2FS1550413116305538%3Fshowall%3Dtrue) |  |  |  | **x** |  |  |  | **x** |  |  |  |  |  |
| **19** | [**Prolonged transfer of feces from the lean mice modulates gut microbiota in obese mice**](https://link.springer.com/article/10.1186/s12986-016-0116-8) | **x** |  |  | **x** |  |  |  |  |  |  |  |  |  |
| **20** | [**Diet-induced extinctions in the gut microbiota compound over generations**](https://www.nature.com/articles/nature16504) |  | **x** |  |  |  |  |  |  |  |  |  |  |  |
| **21** | [**Unusual sub-genus associations of faecal Prevotella and Bacteroides with specific dietary patterns**](https://microbiomejournal.biomedcentral.com/articles/10.1186/s40168-016-0202-1) |  |  | **x** |  |  |  |  |  |  |  |  |  |  |
| **22** | [**Ethnic diversity in infant gut microbiota is apparent before the introduction of complementary diets**](https://www.tandfonline.com/doi/full/10.1080/19490976.2020.1756150) |  | **x** |  |  |  |  |  |  |  |  |  |  |  |
| **23** | [**Persistent gut microbiota immaturity in malnourished Bangladeshi children**](https://www.nature.com/articles/nature13421) |  | **x** |  |  |  |  |  |  |  |  |  |  |  |
| **24** | [**The effect of legume supplementation on the gut microbiota in rural Malawian infants aged 6 to 12 months**](https://academic.oup.com/ajcn/article-abstract/111/4/884/5734257) |  |  |  |  |  |  |  |  | **x** |  |  |  |  |
| **25** | [**The Pervasive Effects of an Antibiotic on the Human Gut Microbiota, as Revealed by Deep 16S rRNA Sequencing**](https://journals.plos.org/plosbiology/article?id=10.1371/journal.pbio.0060280) |  |  |  |  | **x** |  |  |  |  |  |  |  |  |
| **26** | [**Impact of diet in shaping gut microbiota revealed by a comparative study in children from Europe and rural Africa**](https://www.pnas.org/content/107/33/14691) | **x** | **x** |  |  |  |  |  |  |  |  |  |  |  |
| **27** | [**Impact of Westernized Diet on Gut Microbiota in Children on Leyte Island**](https://pubmed.ncbi.nlm.nih.gov/28261164/) | **x** |  | **x** |  |  |  |  |  |  |  |  |  |  |
| **28** | [**Gut Microbiota and Dietary Intake of Normal-Weight and Overweight Filipino Children**](https://www.mdpi.com/2076-2607/8/7/1015) | **x** | **x** |  |  |  |  |  |  |  |  |  |  |  |
| **29** | [**Diversity in gut bacterial community of school-age children in Asia**](https://www.ncbi.nlm.nih.gov/pmc/articles/PMC4336934/#s1) |  | **x** | **x** |  |  |  |  |  |  |  |  |  |  |
| **30** | [**Environmental exposures and child and maternal gut microbiota in rural Malawi**](https://onlinelibrary.wiley.com/doi/full/10.1111/ppe.12623) |  | **x** |  |  |  |  |  |  |  |  |  |  |  |
| **31** | [**Population structure of human gut bacteria in a diverse cohort from rural Tanzania and Botswana**](https://genomebiology.biomedcentral.com/articles/10.1186/s13059-018-1616-9#MOESM2) |  | **x** |  |  |  |  |  |  |  |  |  |  |  |
| **32** | [**Response of the human gut and saliva microbiome to urbanization in Cameroon**](https://www.nature.com/articles/s41598-020-59849-9) |  |  | **x** | **x** | **x** | **x** |  |  | **x** |  |  |  |  |
| **33** | [**Response of the human gut and saliva microbiome to urbanization in Cameroon**](https://www.nature.com/articles/s41598-020-59849-9) |  | **x** |  |  |  |  |  |  |  |  |  |  |  |
| **34** | [**Seasonal cycling in the gut microbiome of the Hadza hunter-gatherers of Tanzania**](https://www.science.org/doi/abs/10.1126/science.aan4834) | **x** | **x** | **x** |  |  |  |  |  |  |  |  |  |  |
| **35** | [**Age-related changes in gut microbiota composition from newborn to centenarian: a cross-sectional study**](https://pubmed.ncbi.nlm.nih.gov/27220822/) | **x** | **x** | **x** |  |  |  |  |  |  |  |  |  |  |
| **36** | [**Diet Quality, Food Groups and Nutrients Associated with the Gut Microbiota in a Nonwestern Population**](https://www.mdpi.com/2072-6643/12/10/2938?utm_source=TrendMD&utm_medium=cpc&utm_campaign=Nutrients_TrendMD_0) |  | **x** | **x** |  |  |  |  |  |  |  |  |  |  |
| **37** | [**Gut Microbiota Bacterial Species Associated with Mediterranean Diet-Related Food Groups in a Northern Spanish Population**](https://www.mdpi.com/2072-6643/13/2/636/htm) |  | **x** |  |  |  |  |  |  |  |  |  |  |  |
| **38** | [**Influence of habitual dietary fibre intake on the responsiveness of the gut microbiota to a prebiotic: protocol for a randomised, double-blind, placebo-controlled, cross-over, single-centre study**](https://pubmed.ncbi.nlm.nih.gov/27591024/) | **x** | **x** | **x** | **x** |  | **x** |  |  | **x** |  |  |  |  |
| **39** | [**Diversity in gut bacterial community of school-age children in Asia**](https://www.nature.com/articles/srep08397) |  | **x** | **x** |  |  |  |  |  | **x** |  |  |  |  |
| **40** | [**Taxonomic Composition and Diversity of the Gut Microbiota in Relation to Habitual Dietary Intake in Korean Adults**](https://www.mdpi.com/2072-6643/13/2/366) |  | **x** | **x** |  |  |  |  |  | **x** |  |  |  |  |
| **41** | [**Developmental trajectory of the healthy human gut microbiota during the first 5 years of life**](https://www.sciencedirect.com/science/article/pii/S1931312821001001) |  | **x** | **x** |  |  |  |  |  | **x** |  |  |  |  |
| **42** | [**Effects of dietary fat on gut microbiota and faecal metabolites, and their relationship with cardiometabolic risk factors: a 6-month randomised controlled-feeding trial**](https://gut.bmj.com/content/68/8/1417.abstract) | **x** | **x** |  |  |  |  | **x** |  |  |  |  |  |  |
| **43** | [**Ethnic diversity in infant gut microbiota is apparent before the introduction of complementary diets**](https://gut.bmj.com/content/68/8/1417.abstract) |  | **x** |  |  |  |  |  |  |  |  |  |  |  |
| **44** | [**US Immigration Westernizes the Human Gut Microbiome**](https://pubmed.ncbi.nlm.nih.gov/30388453/) |  | **x** |  |  |  |  |  |  | **x** |  |  |  |  |
| **45** | [**Evolution of gut microbiota composition from birth to 24 weeks in the INFANTMET Cohort**](https://pubmed.ncbi.nlm.nih.gov/28095889/) |  | **x** |  |  |  |  |  |  |  |  |  |  |  |
| **46** | [**Impact of a Moderately Hypocaloric Mediterranean Diet on the Gut Microbiota Composition of Italian Obese Patients**](https://www.ncbi.nlm.nih.gov/pmc/articles/PMC7551852/) |  | **x** |  |  |  |  |  |  |  |  |  |  |  |
| **47** | [**Microbiome Responses to an Uncontrolled Short-Term Diet Intervention in the Frame of the Citizen Science Project**](https://www.mdpi.com/2072-6643/10/5/576) | **x** | **x** |  |  |  |  |  |  |  |  |  |  |  |
| **48** | [**Dynamics of Human Gut Microbiota and Short-Chain Fatty Acids in Response to Dietary Interventions with Three Fermentable Fibers**](https://www.ncbi.nlm.nih.gov/pmc/articles/PMC6355990/) |  |  |  | **x** |  |  |  |  |  |  |  |  |  |
| **49** | [**Gut microbiota plasticity is correlated with sustained weight loss on a low-carb or low-fat dietary intervention**](https://www.nature.com/articles/s41598-020-58000-y) |  |  |  |  |  |  |  |  | **x** |  |  |  |  |
| **50** | [**American Gut: an Open Platform for Citizen Science Microbiome Research**](https://journals.asm.org/doi/full/10.1128/mSystems.00031-18) |  |  |  |  |  |  |  |  | **x** |  |  |  |  |
| **51** | [**Gut-microbiota-targeted diets modulate human immune status**](https://www.sciencedirect.com/science/article/abs/pii/S0092867421007546) |  | **x** | **x** |  |  |  |  |  | **x** |  |  |  |  |
| **52** | [**Differential Analysis of Hypertension-Associated Intestinal Microbiota**](https://www.medsci.org/v16p0872.htm) | **x** | **x** |  | **x** |  |  | **x** |  |  |  |  |  |  |
| **53** | [**Gut Microbiota and Fecal Levels of Short-Chain Fatty Acids Differ Upon 24-Hour Blood Pressure Levels in Men**](https://www.ahajournals.org/doi/full/10.1161/HYPERTENSIONAHA.118.12588) | **x** |  |  | **x** |  |  |  |  |  |  |  |  |  |
| **54** | [**Gut microbiota associations with common diseases and prescription medications in a population-based cohort**](https://www.nature.com/articles/s41467-018-05184-7) |  | **x** | **x** |  |  |  |  |  | **x** |  |  |  |  |
| **55** | [**Gut microbiota diversity after autologous fecal microbiota transfer in acute myeloid leukemia patients**](https://www.ncbi.nlm.nih.gov/pmc/articles/PMC8149453/) |  | **x** |  | **x** |  |  |  |  |  |  |  |  |  |
| **56** | [**Altered fecal microbiota composition in patients with major depressive disorder**](https://pubmed.ncbi.nlm.nih.gov/25882912/) | **x** | **x** |  | **x** |  |  | **x** |  |  |  |  |  |  |
| **57** | [**Possible association of Firmicutes in the gut microbiota of patients with major depressive disorder**](https://pubmed.ncbi.nlm.nih.gov/30584306/) | **x** | **x** |  |  |  |  | **x** |  | **x** |  |  |  |  |
| **58** | [**Shotgun metagenomics reveals both taxonomic and tryptophan pathway differences of gut microbiota in major depressive disorder patients**](https://pubmed.ncbi.nlm.nih.gov/31685046/) |  | **x** |  |  |  |  |  |  |  | **x** |  |  |  |
| **59** | [**Similarly in depression, nuances of gut microbiota: Evidences from a shotgun metagenomics sequencing study on major depressive disorder versus bipolar disorder with current major depressive episode patients**](https://pubmed.ncbi.nlm.nih.gov/30927646/) | **x** | **x** |  | **x** |  |  |  |  |  |  | **x** |  |  |
| **60** | [**Age-specific differential changes on gut microbiota composition in patients with major depressive disorder**](https://www.aging-us.com/article/102775) | **x** |  |  |  |  |  | **x** |  |  |  |  |  |  |
| **61** | [**Shotgun metagenomics reveals both taxonomic and tryptophan pathway differences of gut microbiota in bipolar disorder with current major depressive episode patients**](https://www.sciencedirect.com/science/article/abs/pii/S0165032720327051) |  | **x** |  | **x** |  |  |  |  |  | **x** |  |  |  |
| **62** | [**Functional microbiome deficits associated with ageing: Chronological age threshold**](https://www.ncbi.nlm.nih.gov/pmc/articles/PMC6974723/) |  | **x** |  |  |  |  |  |  |  |  |  | **x** | **x** |
| **63** | [**Associations of physical activity with gut microbiota in pre-adolescent children**](https://www.ncbi.nlm.nih.gov/pmc/articles/PMC8843867/) |  |  | **x** |  |  |  |  |  | **x** |  |  | **x** |  |
| **64** | [**Comparative Studies of the Gut Microbiota in the Offspring of Mothers With and Without Gestational Diabetes**](https://www.ncbi.nlm.nih.gov/pmc/articles/PMC7645212/) |  | **x** | **x** |  |  |  |  |  |  |  |  | **x** |  |
| **65** | [**Characteristics and Mediating Effect of Gut Microbiota With Experience of Childhood Maltreatment in Major Depressive Disorder**](https://www.ncbi.nlm.nih.gov/pmc/articles/PMC9238290/) | **x** | **x** | **x** | **x** |  |  |  |  | **x** |  |  | **x** |  |
| **66** | [**The Gut Microbiota Profile According to Glycemic Control in Type 1 Diabetes Patients Treated with Personal Insulin Pumps**](https://www.ncbi.nlm.nih.gov/pmc/articles/PMC7826603/) | **x** | **x** | **x** | **x** |  |  |  |  |  |  |  | **x** |  |
| **67** | [**Alterations of gut microbiome in patients with type 2 diabetes mellitus who had undergone cholecystectomy**](https://journals.physiology.org/doi/full/10.1152/ajpendo.00471.2020?rfr_dat=cr_pub++0pubmed&url_ver=Z39.88-2003&rfr_id=ori%3Arid%3Acrossref.org) | **x** | **x** |  | **x** |  |  |  |  |  |  |  | **x** |  |
| **68** | [**Effects of HIV, antiretroviral therapy and prebiotics on the active fraction of the gut microbiota**](https://journals.lww.com/aidsonline/Fulltext/2018/06190/Effects_of_HIV,_antiretroviral_therapy_and.3.aspx) |  | **x** |  |  |  |  |  |  |  |  |  | **x** | **x** |

**Table S2.** Metadata of the 13 public human microbiota experimental studies used to perform the selected alpha metrics. Studies are referenced according to the column “Code.” Column “Groups” contain experimental design information. Column “doi” contains the link to the online version of the paper. The total number of samples from the combined analyzed projects in this study is 4,596.

| **Code** | **Title** | **Year** | **doi** | **16S Region** | **Groups** | **Number of samples** | **Longitudinal?** |
| --- | --- | --- | --- | --- | --- | --- | --- |
| 147_volunteers | Response of the human gut and saliva microbiome to urbanization in Cameroon | 2020 | [10.1038/s41598-020-59849-9](https://doi.org/10.1038%2Fs41598-020-59849-9) | V4 | rural (79)  semiurban (32)  urban (32) | 143 | NO |
|  |  |  |  |  |  |  |  |
| 303_asian | Diversity in gut bacterial community of school-age children in Asia | 2015 | [10.1038/srep08397](https://doi.org/10.1038%2Fsrep08397) | V6-V8 | Japan (83)  China (59)  Indonesia (55)  Taiwan (53)  Thailand (52) | 302 | NO |
|  |  |  |  |  |  |  |  |
| 34_L_H_fibre | Influence of habitual dietary fibre intake on the responsiveness of the gut microbiota to a prebiotic: protocol for a randomized, double-blind, placebo-controlled, cross-over, single-center study | 2016 | [10.1136/bmjopen-2016-012504](https://doi.org/10.1136%2Fbmjopen-2016-012504) | V3-V4 | Fiber_H_pre (20)  Fiber_H_post (20)  Fiber_L_pre (14)  Fiber_L_post (14) | 40 | YES |
|  |  |  |  |  |  |  |  |
|  |  |  |  |  |  | 28 | YES |
|  |  |  |  |  |  |  |  |
| 514_IMP_project | US Immigration Westernizes the Human Gut Microbiome | 2019 | [10.1016/j.cell.2018.10.029](https://doi.org/10.1016%2Fj.cell.2018.10.029) | V4 | Thailand (179)  Th->USA (281)  USA (54)  CtrL (36) | 235 | NO |
|  |  |  |  |  |  |  |  |
| 23_obese | Impact of a Moderately Hypocaloric Mediterranean Diet on the Gut Microbiota Composition of Italian Obese Patients | 2020 | [10.3390/nu12092707](https://doi.org/10.3390%2Fnu12092707) | V3 - V4 | BL (23)  3M (23) | 46 | YES |
|  |  |  |  |  |  |  |  |
| 248_citizen | Microbiome Responses to an Uncontrolled Short-Term Diet Intervention in the Frame of the Citizen Science Project | 2018 | [10.3390/nu10050576](https://doi.org/10.3390%2Fnu10050576) | V4 | Before (265)  After (246) | 511 | YES |
|  |  |  |  |  |  |  |  |
| 153_italian | Unusual sub-genus associations of fecal Prevotella and Bacteroides with specific dietary patterns | 2016 | [10.1136/gutjnl-2015-309957](https://doi.org/10.1136/gutjnl-2015-309957) | V1 - V3 | Omnivore (47)  Vegetarian (50)  Vegan (53) | 99 | NO |
|  |  |  |  |  |  |  |  |
| 95_obese | Link between gut microbiota and health outcomes in inulin -treated obese patients: Lessons from the Food4Gut multicenter randomized placebo-controlled trial | 2020 | [10.1016/j.clnu.2020.04.005](https://doi.org/10.1016/j.clnu.2020.04.005) | V5 - V6 | Prebiotic_BL (48)  Placebo_BL (47)  Prebiotic_3M (48)  Placebo_3M (47) | 96 | YES |
|  |  |  |  |  |  |  |  |
| 29_children_EU_Africa | Impact of diet in shaping gut microbiota revealed by a comparative study in children from Europe and rural Africa | 2010 | [10.1073/pnas.1005963107](https://doi.org/10.1073/pnas.1005963107) | V5 - V6 | Eu (15)  Af (14) | 29 | NO |
|  |  |  |  |  |  |  |  |
| 471_swedish | Developmental trajectory of the healthy human gut microbiota during the first 5 years of life | 2021 | [10.1016/j.chom.2021.02.021](https://doi.org/10.1016/j.chom.2021.02.021) | V4 | M (357)  NB (246)  4M (412)  12M (397)  3Y (336)  5Y (288) | 534 | YES |
|  |  |  |  |  |  |  |  |
| 161_LCarb_and_LFat | Gut microbiota plasticity is correlated with sustained weight loss on a low-carb or low-fat dietary intervention | 2020 | [10.1038/s41598-020-58000-y](https://doi.org/10.1038%2Fs41598-020-58000-y) | V4 | BL_VS (52)  10W_VS (46) | 98 | YES |
|  |  |  |  |  |  |  |  |
| 888_AGP | American Gut: an Open Platform for Citizen Science Microbiome Research | 2018 | [10.1128/mSystems.00031-18](https://doi.org/10.1128/msystems.00031-18) | V4 | several conditions | 2446 | NO |
|  |  |  |  |  |  |  |  |
|  |  |  |  |  |  |  |  |
|  |  |  |  |  |  |  |  |
| 18_mixed | Gut-microbiota-targeted diets modulate human immune status | 2021 | [10.1016/j.cell.2021.06.019](https://doi.org/10.1016/j.cell.2021.06.019) | V4 | High Fiber (18)  High Fermented (18) | 311 | YES |

**Figure S2**: Scatter plot of singletons and observed features (y axis) by sequencing depth (x axis).


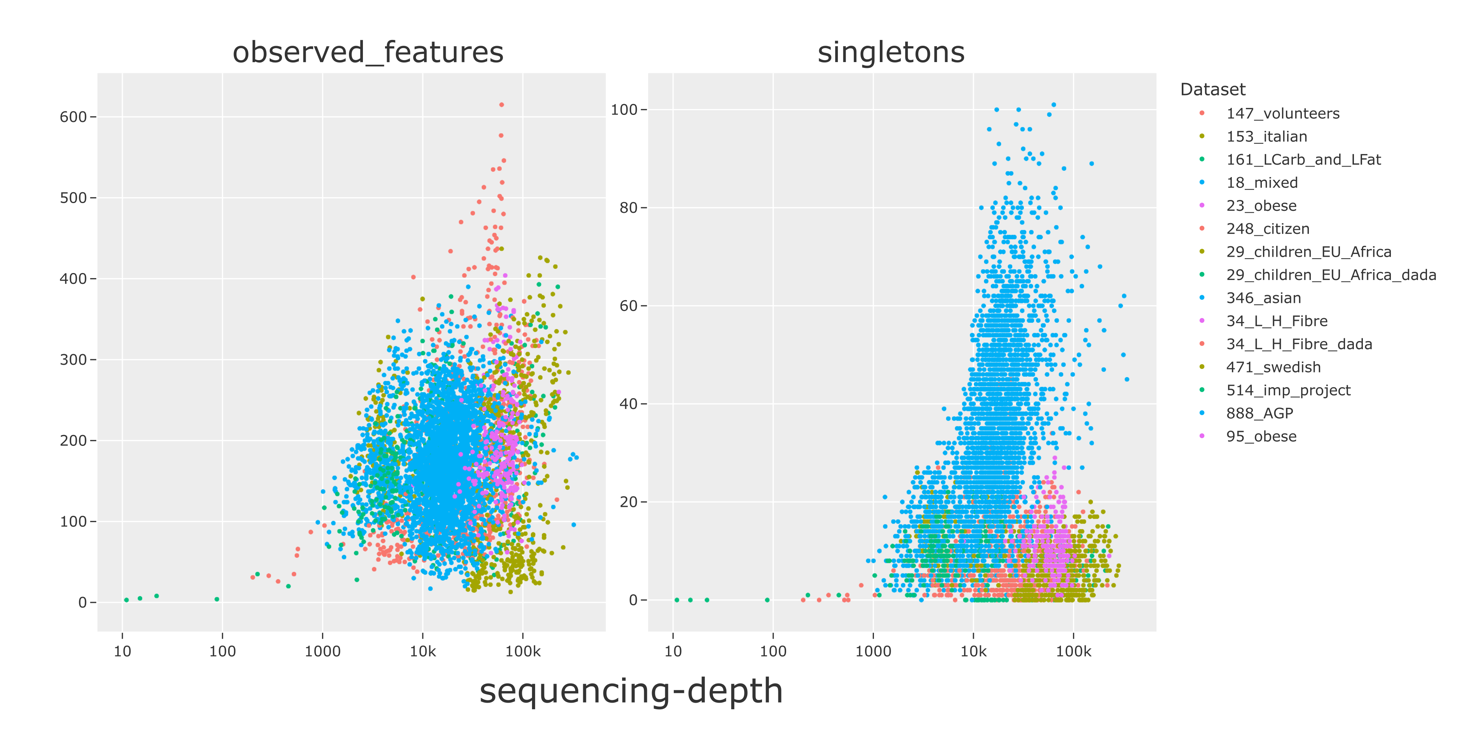


**Table S3**: Spearman correlation coefficients calculated for observed features and singletons, correlating them with sequencing depth. In both cases there was no strong correlation.

|  | Spearman statistics | p_value |
| --- | --- | --- |
| Observed features | -0.08689713980941335 | 3.239366005700848e-10 |
| Singletons | 0.1358227357753221 | 6.609560207924828e-23 |

**Figure S3**: Boxplots for total observed features and singletons for each used 16S amplicon.


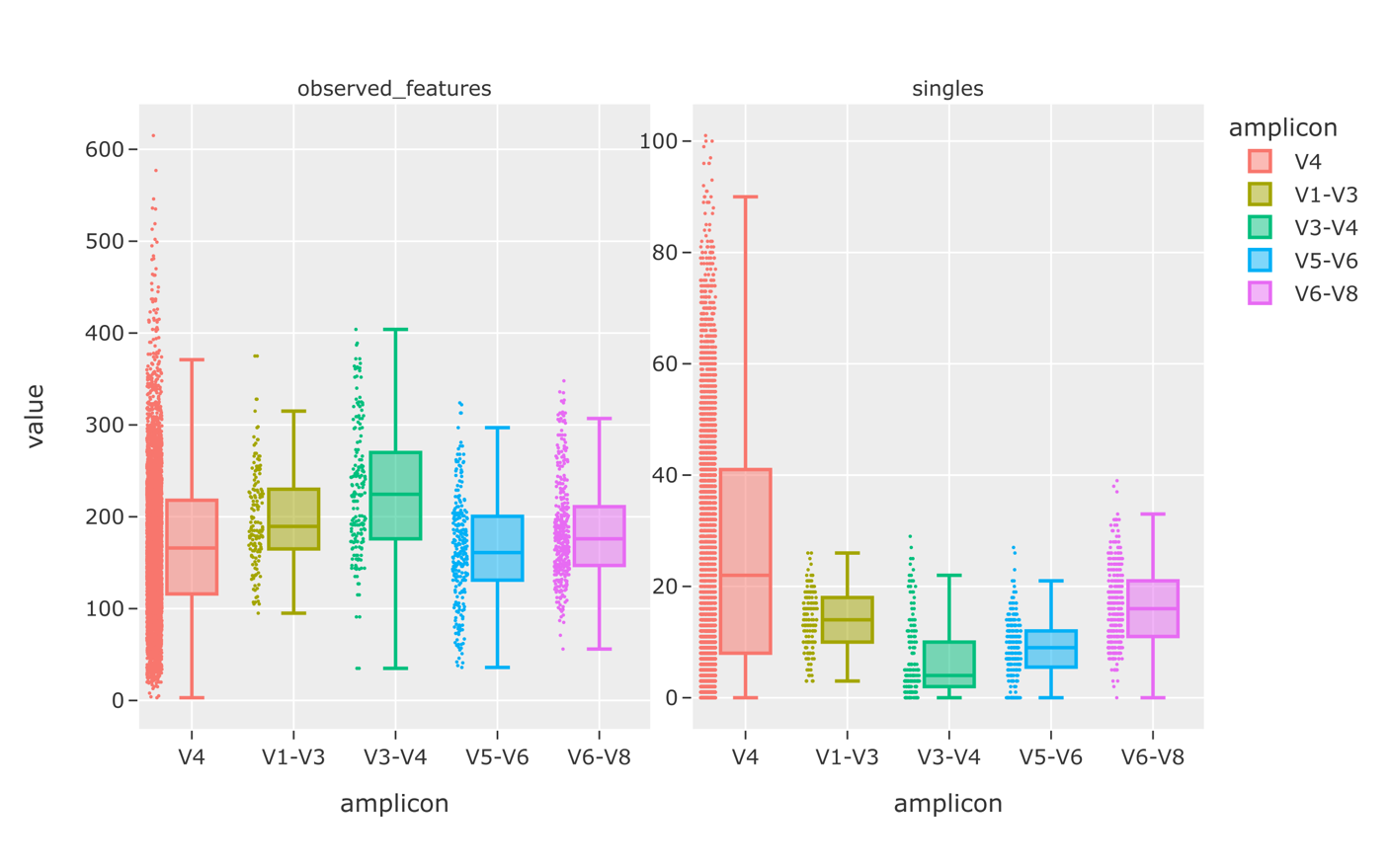


**Table S4**: Means and standard deviations for all 16S amplicon regions amplified in the used datasets. Kruskal-Wallis statistics and matching p-value are shown. Kruskal-Wallis was used to validate differences in the means between the different 16S amplicon regions, as the data did not meet the normal distribution necessary for ANOVA testing.

|  | Kruskal-Wallis Statistics | p_value |
| --- | --- | --- |
| Observed_features | 146.16895071059517 | 1.347543675472802e-30 |
| Singletons | 401.8067948600021 | 1.1321571859196657e-85 |

|  | Observed_features | | Singles | |
| --- | --- | --- | --- | --- |
| Amplicon | mean | std | mean | std |
| V1-V3 | 199.753333 | 53.994523 | 13.806667 | 5.210594 |
| V3-V4 | 229.483516 | 70.915616 | 6.664835 | 6.766245 |
| V4 | 171.098578 | 76.816570 | 25.766022 | 20.539865 |
| V5-V6 | 163.508065 | 58.883814 | 8.798387 | 5.261548 |
| V6-V8 | 184.919075 | 55.034317 | 16.632948 | 6.909432 |

**Figure S5.** Scatter plot that shows the relationship between Berger Parker metric and the proportion between the most dominant microbe and the second most dominant microbe, applied on the 4,596 samples.


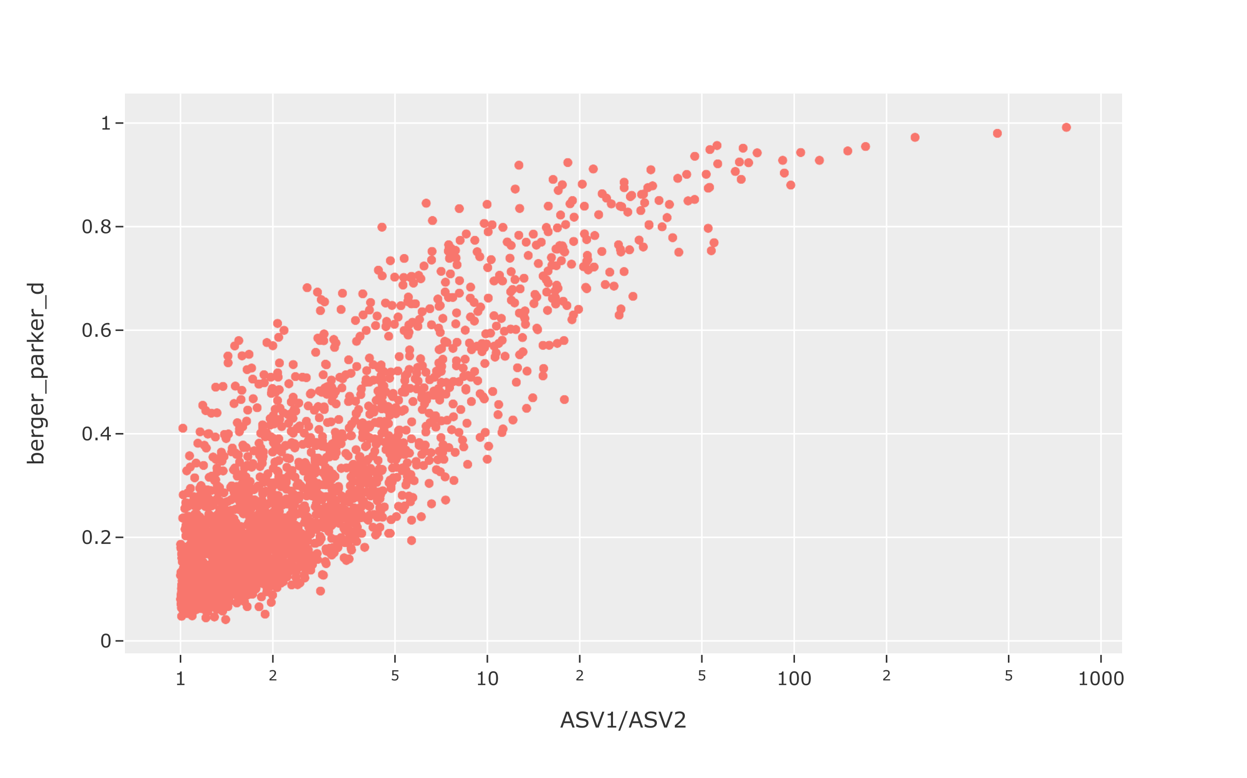


Extreme values of the proportion between the most dominant microbe and the second most dominant microbe metric singled out samples where one microorganism was observed 100x over the next most abundant one. These samples were easiest to identify by this metric and while they did have a reduced number of ASVs they showed no other relevant features in their frequency distributions.

**Figure S5.** Correlation between Shannon, Berger Parker and Observed_features of 4,596 samples. The upper diagonal displays the linear correlation coefficients calculated using Pearson's method, with the R^2^ and p values for each correlation shown in red on the upper left side. The Spearman correlation coefficients (ρ\rho) and their corresponding p values are presented in blue at the upper right corner.


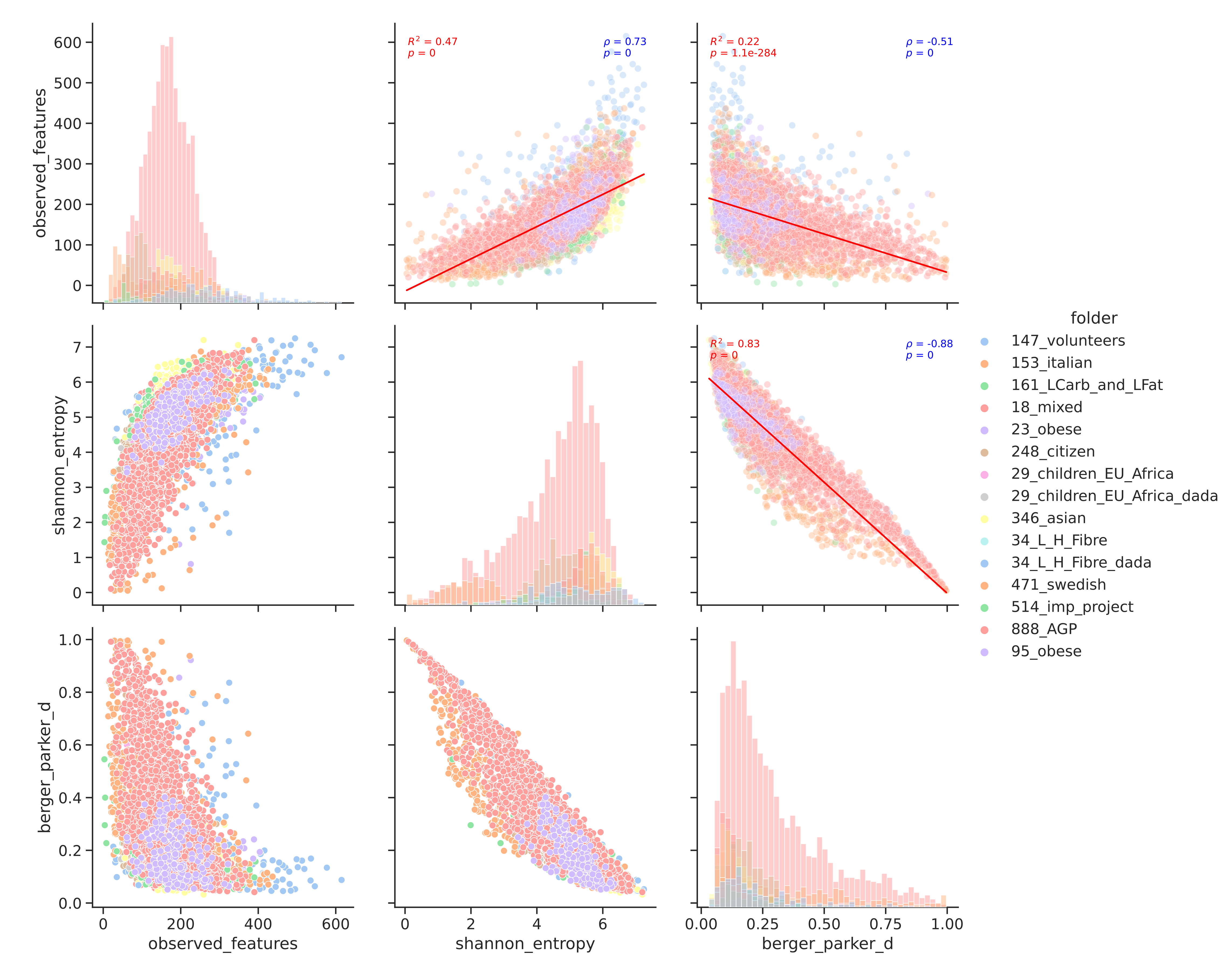


**Supp Figure S6**. Box plot of the distribution of alpha diversity metrics when applied to synthetic datasets. Each box contains the data for the alpha diversity metric displayed on top. The Y axis is the value of the metric, and the X axis is categorical, with one box per dataset.


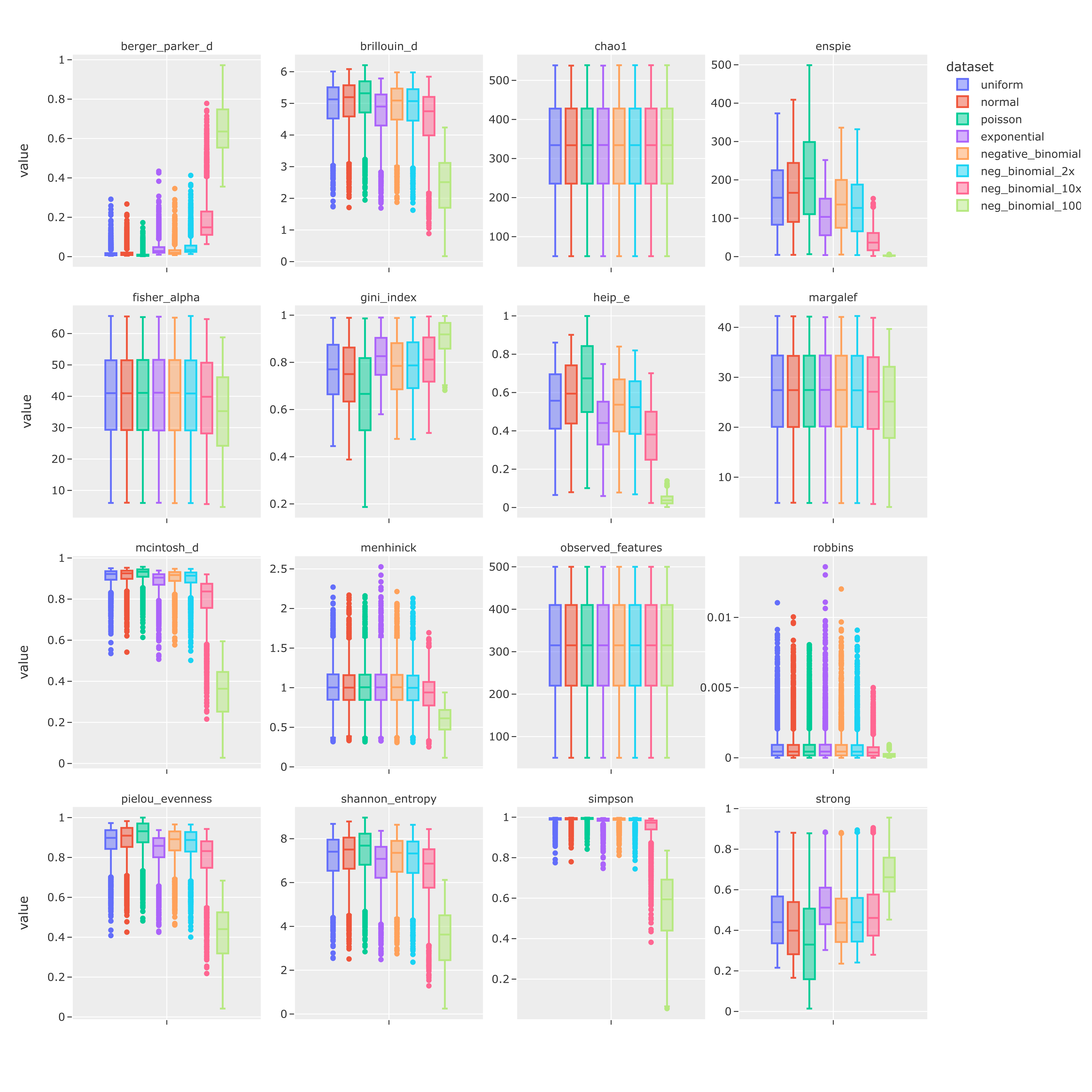


**Supp. Figure S7.** Relationship between singletons (Y axis) and the number of ASVs (X axis) of the samples in each of the synthetic datasets. Points represent samples, with each plot colored according to a normalized value (proportion) of the corresponding alpha diversity metric. Plots are organized bymetrics in the rows and datasets in the columns.


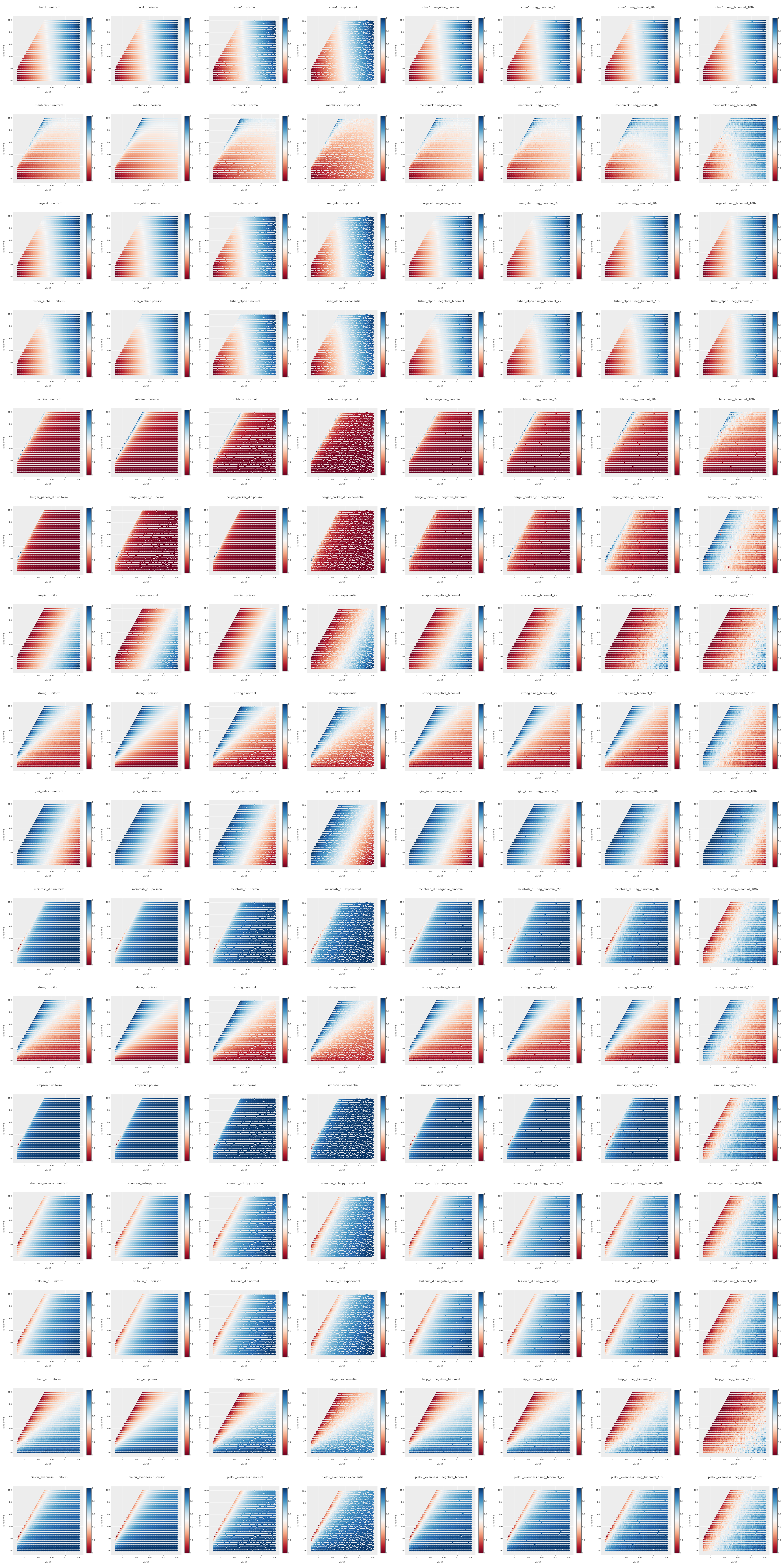

Supplement: Supplementary file 1 — Supplementary Material 1 [file 41598_2024_77864_MOESM1_ESM.docx]
